# Passable: An Intelligent Traffic Light System with Integrated Incident Detection and Vehicle Alerting

**DOI:** 10.3390/s25185760

**Published:** 2025-09-16

**Authors:** Ohoud Alzamzami, Zainab Alsaggaf, Reema AlMalki, Rawan Alghamdi, Amal Babour, Lama Al Khuzayem

**Affiliations:** 1 Department of Computer Science, Faculty of Computing and Information Technology, King Abdulaziz University, Jeddah 21589, Saudi Arabia; zalialsaggaf@stu.kau.edu.sa (Z.A.); rsalemalmalki@stu.kau.edu.sa (R.A.); rsaeedalghamdi0007@stu.kau.edu.sa (R.A.); lalkhuzayem@kau.edu.sa (L.A.K.); 2 Department of Information Systems, Faculty of Computing and Information Technology, King Abdulaziz University, Jeddah 21589, Saudi Arabia; ababor@kau.edu.sa

**Keywords:** intelligent transportation systems, adaptive traffic light, Internet of Things, computer vision, deep learning

## Abstract

The advancement of Artificial Intelligence (AI) and the Internet of Things (IoT) has accelerated the development of Intelligent Transportation Systems (ITS) in smart cities, playing a crucial role in optimizing traffic flow, enhancing road safety, and improving the driving experience. With urban traffic becoming increasingly complex, timely detection and response to congestion and accidents are critical to ensuring safety and situational awareness. This paper presents Passable, an intelligent and adaptive traffic light control system that monitors traffic conditions in real time using deep learning and computer vision. By analyzing images captured from cameras at traffic lights, Passable detects road incidents and dynamically adjusts signal timings based on current vehicle density. It also employs wireless communication to alert drivers and update a centralized dashboard accessible to traffic management authorities. A working prototype integrating both hardware and software components was developed and evaluated. Results demonstrate the feasibility and effectiveness of designing an adaptive traffic signal control system that integrates incident detection, instantaneous communication, and immediate reporting to the relevant authorities. Such a design can enhance traffic efficiency and contribute to road safety. Future work will involve testing the system with real-world vehicular communication technologies on multiple coordinated intersections while integrating pedestrian and emergency vehicle detection.

## 1. Introduction

The increasing number of vehicles on the road around the world has intensified congestion, driving up fuel consumption, increasing carbon dioxide (CO_2_) emissions, and increasing accident risks [1,2]. Every year, more than 1.35 million people lose their lives in road traffic accidents, representing approximately 2.2% of global deaths [3]. In Saudi Arabia, traffic accidents are one of the leading causes of death and disability, with annual losses estimated at USD 16 billion (approximately SAR 60 billion) [4,5]. Secondary accidents, triggered by driver distraction or sudden maneuvers near a primary crash, further exacerbate congestion and increase the severity of the injury. Beyond the human toll, road crashes impose significant socio-economic costs, including healthcare expenses, property damage, and lost productivity. These wide-ranging impacts underscore the urgency of adopting intelligent transportation initiatives that enhance both safety and environmental sustainability [6].

Urban intersections are among the most critical and hazardous points in road networks. Their complex layouts, involving multiple lanes, signal phases, and conflicting traffic flows, demand heightened attention from all road users. A large-scale analysis of over 10 billion traffic observations revealed a non-linear causal relationship between congestion and accident frequency [7], underscoring the urgent need to enhance both safety and mobility at intersections.

Traditional traffic light systems, typically operating on fixed schedules, are ill-suited for dynamic traffic conditions. Manual adjustments are often required, leading to longer delays, higher emissions, and elevated accident risk. To address these limitations, researchers have proposed intelligent traffic control systems that leverage Artificial Intelligence (AI) and the Internet of Things (IoT) for adaptive and efficient traffic management [8,9,10,11,12,13,14,15,16,17].

In smart cities, well-managed urban transportation networks are essential for road safety, reduced congestion, shorter travel times, economic growth, and environmental sustainability [18,19]. While AI techniques have been applied extensively to either traffic congestion detection [20,21,22,23,24,25] or accident detection [26,27,28,29,30,31,32], most intelligent traffic light systems address these issues separately. This separation is problematic because congestion and accidents are closely interrelated—each can worsen the impact of the other [33,34].

Only a few existing solutions integrate both congestion and accident detection while actively notifying drivers [35]. To bridge this gap, we propose Passable, an intelligent traffic light control system, translated as “Salik” in Arabic, that employs deep learning and computer vision techniques to unify incident detection, adaptive signal control, driver communication, and centralized monitoring. Unlike previous studies, Passable integrates the following contributions in a fully functional adaptive traffic light system:

- Detects congestion and accidents simultaneously using AI-based computer vision.
- Adjusts green time adaptively based on real-time traffic conditions.
- Alerts drivers via in-vehicle wireless communication.
- Provides centralized monitoring through a cloud-integrated dashboard.

Although individual components are based on established methods, their combined implementation and synchronization in a fully integrated and functional prototype tailored to urban traffic conditions offer a practical advancement in the deployment of intelligent transportation.

The remainder of this paper is organized as follows. Section 2 reviews related work on congestion detection, accident detection, and adaptive traffic control. Section 3 introduces the proposed system framework. Section 4 and Section 5 describe the data collection methods and system design. Section 6 and Section 7 detail the deep learning models and system software and hardware implementation. Section 8 presents the Passable system testing and analytical evaluation. Section 9 discusses the system performance and limitations, highlighting directions for future work. Finally, Section 10 presents the conclusions.

## 2. Related Work

With the increasing number of vehicles on the roads, traffic congestion and road accidents have become significant challenges to urban mobility. To address these issues, sensors and computer vision-based techniques have been widely adopted in intelligent traffic surveillance and management, particularly for detecting congestion and accidents. Additionally, the development of intelligent and adaptive traffic light control systems has attracted significant interest from the research community. The following subsections review existing studies on Intelligent Transportation Systems.

### 2.1. Vehicle Accident Detection

Accident detection has been explored through both sensor-based and vision-based approaches. A framework for accident detection using multimodal in-vehicle sensors combined with machine learning techniques was developed [26]. The system extracted features using a Convolutional Neural Network (CNN) and classified them using a Support Vector Machine (SVM). Incorporating data from multiple sensor channels improved detection performance. However, challenges such as limited dataset availability and variability in sensor types hindered generalization and complicated comparisons with other models.

Vision-based systems, which utilize traffic images, have become prominent due to their scalability and cost-effectiveness. In [27], a method that employed a Gaussian Mixture Model (GMM) to separate foreground and background in video frames was proposed. Vehicles were tracked using the mean shift algorithm, and features such as position, direction, and acceleration were used to make the final decision regarding accident detection. Simulations confirmed the potential of this method for real-time detection.

In another study, a system was proposed to detect accidents and transmit relevant information to emergency vehicles [28]. It utilized the “You Only Look Once” (YOLOv3) algorithm for recognizing various types of accidents, including those caused by animals. Integration with a mobile application enabled timely notifications for emergency personnel.

Deep learning techniques were applied in another study for accident detection and localization [29]. Data were gathered from dash camera footage on YouTube videos. The results of the study achieved a score of 84.70% for Area Under the Curve (AUC). The proposed model outperformed the state-of-the-art unsupervised learning-based approach by 11.7%.

An accident detection system utilizing YOLOv5 for real-time vehicle detection using Closed-Circuit Television (CCTV) images was proposed in [30]. A customized dataset of 1000 annotated photos, captured under various conditions (such as rain or poor visibility), was used to train the system. The system detects potential accidents and classifies vehicles into four categories. The CNN-based YOLOv5 model showed improved precision and accuracy compared to other state-of-the-art methods. However, the system lacks functionality for classifying accident severity or issuing alerts to nearby vehicles.

Advanced hybrid frameworks have emerged to improve accident prediction in complex traffic scenarios. For example, a vision-based real-time accident prediction framework using deep neural networks was proposed in [31]. The framework integrates motion temporal templates and fuzzy time-slicing. The system achieved a high detection rate of 98.5%, outperforming existing methods.

Another hybrid deep learning approach for detecting traffic accidents from video frames was proposed in [32]. The method employed YOLOv5 for dynamic object detection and background reduction to eliminate extraneous information, followed by a CNN encoder to extract spatial features and a Transformer decoder to capture temporal correlations between video frames. This model enabled concurrent frame analysis and achieved an accuracy of 96% on the Car Crash Dataset. This approach outperforms traditional models in complicated traffic conditions by increasing accuracy and processing efficiency.

### 2.2. Vehicle Congestion Detection

Advancements in congestion detection have been driven by IoT- and VANET-based approaches, vision-based methods, and machine learning-driven predictive models. IoT-enabled congestion detection systems often rely on connected vehicles and vehicular ad hoc networks (VANETs) to collect and transmit traffic data. For instance, the authors in [20] proposed a system that integrates big data, VANETs, and both infrastructure-to-infrastructure (I2I) and vehicle-to-infrastructure (V2I) communication to detect congestion and suggest alternative routes to drivers. The system architecture incorporated a Cassandra-based big data cluster, OMNeT++ for event-based simulation, SUMO for traffic modeling, and the Veins framework for vehicular networking. Experimental results showed that the system effectively reduced travel time and CO_2_ emissions through real-time congestion detection and rerouting.

Similarly, the authors in [21] developed a big data-based framework for detecting congestion patterns from VANET data. The approach used vehicle coordinates and timestamps to identify congestion hotspots and peak intervals. Mobility modeling was performed using SUMO, while Hadoop facilitated large-scale data processing. Although the system showed promising results in detecting dense traffic conditions, scalability challenges were noted, particularly for deployment in large environments.

Vision-based systems utilize live video streams or aerial imagery to analyze traffic flow and detect congested areas. In [22], the challenge of detecting small vehicles in aerial images was addressed. By improving the YOLOv3 architecture, the authors enhanced multiscale feature fusion and anchor box clustering and added a refined residual module. Their model achieved an accuracy of 89.3% on the VEDAI dataset, significantly outperforming the baseline YOLOv3 model in terms of recall and F1 score.

In another relevant study, a real-time object detection model (SSD with MobileNet) was applied to recognize vehicles and pedestrians [23]. The model, pre-trained on COCO and fine-tuned for PASCAL VOC, achieves 72.8% mAP, successfully detecting objects such as cars and pedestrians in real time with a confidence of 94.93% and 91.34%, respectively. However, its performance declined in high-density-traffic scenarios due to occlusions and false positives, suggesting limitations in vision-based systems under complex conditions.

Several studies have explored predictive congestion detection using machine learning algorithms trained on historical and contextual data. In [24], the authors proposed a data-driven approach incorporating variables such as rainfall, public holidays, weekdays, and time of day. The study compared four models: simple and polynomial linear regression, feedforward neural networks (FFNNs), and radial basis function networks (RBFNs). The FFNN model achieved the best performance in predicting traffic congestion, particularly in minimizing average waiting and movement times.

Although primarily focused on accident detection, a machine learning framework that indirectly supports congestion analysis through road-user detection under low-light conditions was introduced in [25]. The system used YOLOv8 and histogram equalization on the NightOwls dataset, achieving high mAP scores for pedestrians and cyclists. The detection of vulnerable road users in high-density areas has implications for congestion-aware decision-making in autonomous vehicle systems. The paper highlights challenges such as poor weather performance, hardware costs, and integration with legacy systems.

### 2.3. Adaptive Traffic Light Control Systems

The evolution of adaptive traffic light systems has progressed from early wireless communication frameworks and threshold-based signal controls to AI-powered, sensor-integrated, and edge-computing models. Early efforts focused on communication-based simulation and algorithmic control.

In [8], a simulation framework for Intelligent Transportation Systems (ITS) was developed to evaluate an adaptive traffic light system utilizing wireless communication between vehicles and fixed controller nodes. The system dynamically adjusted signals using real-time data from moving vehicles. It incorporated a custom-built discrete-event VANET simulator and modeled driver behavior using the Wiedemann driver behavior model. Testing at two real-world intersections in Bucharest revealed substantial improvements, including a 28.3% reduction in average delay, 6.5% lower fuel consumption, and reduced emissions compared to pre-timed signals.

In a related direction, a genetic algorithm to optimize signal timings based on traffic conditions was proposed [9]. The algorithm incorporated parameters such as queue length, and simulation results showed favorable outcomes when real-time traffic flow was taken into account. However, the system’s effectiveness diminished when this context was excluded, emphasizing the importance of accurate input data in adaptive signal control.

Sensor-based systems then emerged as practical, low-cost alternatives. In [10], a Radio Frequency Identification (RFID)-integrated system with existing traffic lights was introduced to facilitate real-time vehicle data collection. Based on vehicle frequency, signals were dynamically adjusted, and congestion was mitigated by upstream alerts triggered when thresholds were exceeded. The alerts utilize the Global System for Mobile Communications (GSM) and the General Packet Radio Service (GPRS). The cyclic threshold-driven approach of the system reduced time wastage but required careful calibration of frequency limits.

In [11], the authors advanced the concept by deploying Raspberry Pi devices, ultrasonic sensors, and cameras to classify traffic volume into low, medium, or high levels. The duration of the signals was adjusted accordingly using image processing and distance measurements. The system transmitted data to a cloud platform for long-term analysis and response in the event of sensor failure, thereby enhancing its adaptive capabilities.

In [12], a magnetometer-based system was proposed where the green light was activated for directions with at least 30 waiting vehicles, using a First In, First Out (FIFO) scheduling approach. Each side received a fixed 3-min green phase. This simple rule-based model demonstrated effectiveness in medium-density intersections but lacked fine-grained adaptability. The prototype utilizes Arduino wireless communication, and it includes a camera and infrared (IR) sensors.

More recently, research has shifted toward AI-enhanced traffic control using computer vision and machine learning. In [13], a hybrid framework combining YOLOv4 for real-time vehicle detection with XGBoost was proposed to predict optimal green light durations. This system accounted for vehicle types and weather conditions using CCTV input and reduced average waiting time by 32.3% compared to fixed-time signals. Its reliance solely on visual data made it a scalable and cost-effective solution for intelligent traffic management.

In [14], an Urban Traffic Control (UTC) system, optimized for dense urban settings, was developed. Using RFID or Dynamic Spectrum Decision Radio (DSDR) technologies, the system adjusted signal timings based on lane weight, congestion indicators, and vehicle priority. Tested using multi-agent simulation in NetLogo, UTC reduced average waiting time by 25.98% with lane flow control and 34.16% under free-flow conditions while requiring minimal infrastructure changes.

Reinforcement learning (RL) has gained attention for its adaptability in complex environments. In [15], four RL-based strategies were evaluated: an actuated traffic signal controller (M1) as the baseline, a single-agent reinforcement learning model using traffic composition as the state (M2), a single-agent reinforcement learning model using Passenger Car Unit (PCU) as the state (M3), and a multi-agent reinforcement learning model using traffic composition as the state (M4). M4 achieved the best performance, reducing green time wastage and achieving an average waiting time of 80 s, which demonstrates RL’s potential for optimizing signals in heterogeneous and dynamic traffic scenarios.

Integrated sensor–vision systems have emerged to address both congestion and safety concerns. In [35], a multimodal system combining roadside sensors (IR, smoke, microphones) and cameras was proposed. A Density-Based Spatial Clustering of Applications with Noise (DBSCAN) algorithm was used to detect congestion, while accidents were identified through sound and motion events. Traffic light timings were adjusted accordingly, and secure, encrypted vehicle communication was ensured via the SEcure Early Traffic-Related EveNt Detection (SEE-TREND) protocol. This approach uniquely combined congestion and accident management in a single, adaptive system.

Edge-computing-based frameworks offer lightweight, real-time deployment capabilities. The authors in [16] introduced the Dynamic Traffic Light System (DTLS), deploying an optimized YOLOv3 model on Raspberry Pi devices. To support low-latency performance, the model was pruned and quantized. Intra-junction communication was handled via IEEE 802.15.4 Deterministic and Synchronous Multi-channel Extension (DSME), while LoRaWAN enabled inter-junction data sharing. DTLS also supported emergency vehicle prioritization and green corridor functionality. Results showed improvements in latency, power consumption, and network overhead.

The authors in [17] proposed an IoT-enabled system using the YOLO D-NET model and edge computing for vehicle and pedestrian detection from CCTV streams. While the model effectively enabled real-time traffic classification, the authors did not elaborate on how the adaptive signal logic was implemented, leaving its applicability in real-world control uncertain.

Finally, the authors of [36] proposed a system that incorporates Unmanned Aerial Vehicles (UAVs) and Visible Light Communication (VLC) technology into traffic light control systems. The study uses UAVs to capture road images and detect congested locations with YOLOv4. The UAVs then use a VLC to broadcast the detection information to ground-based traffic lights, allowing for automated modifications. UAVs are known for their ability to obtain real-time traffic monitoring data from elevated points, and VLC provides a reliable, high-speed, and energy-efficient communication medium. However, according to the authors, one main challenge of using UAVs in traffic monitoring is their limited energy capacity, restricting their flight time and processing power. Another challenge is maintaining stable line-of-sight communication when using VLC, which is difficult due to the movements of UAVs and other environmental factors such as wind, altitude, and traffic density. These challenges could be addressed by integrating UAV- and VLC-based systems with other ITS infrastructure solutions.

Table 1 shows a summary of reviewed adaptive traffic light control systems. Over time, adaptive traffic light systems have evolved from communication-based rule engines to sophisticated, AI-powered, real-time controllers. With the exception of the study [35], previous research has focused exclusively on congestion detection, overlooking accident detection—despite the close interrelation between the two. Identifying and alerting drivers to one type of incident can help mitigate the other. In addition, all prior studies, except the study [35], have excluded driver alerts from adaptive traffic light control, disregarding the potential advantages of driver awareness in reducing the likelihood and impact of both congestion and accidents. The limited incorporation of driver alerting and communication protocols presents additional challenges to deployment in real urban environments.

Moreover, most prior studies have adopted either a simulation or a hardware prototype, lacking practical implementation strategies suitable for urban environments. Furthermore, none of the compared approaches include a centralized dashboard for real-time traffic monitoring and system administration. Passable, on the other hand, overcomes these limitations by providing a unified hardware prototype that combines congestion detection, accident detection, driver alerts, and centralized monitoring. This comprehensive design demonstrates the feasibility, effectiveness, and interoperability of adaptive traffic light systems in smart city environments.

## 3. Passable System Overview

**Figure 1:**
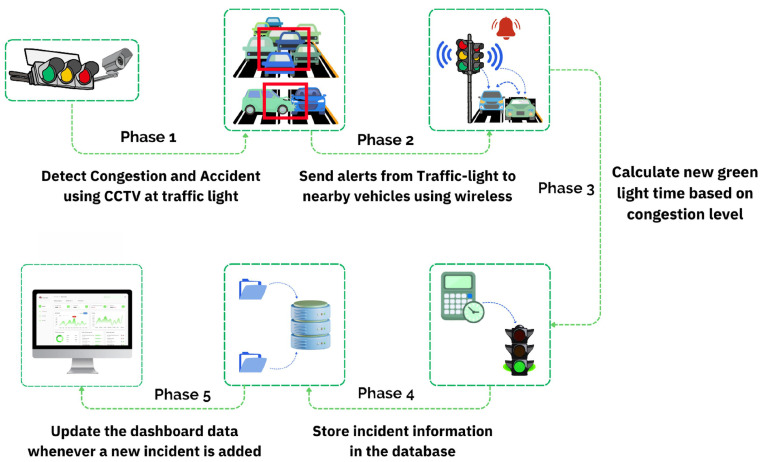
Overview of the Passable system framework integrating hardware, software, AI detection, and wireless communication for adaptive traffic control and incident alerts.

Passable is an intelligent traffic light control system. It relies on computer vision for real-time detection of road incidents, including accidents and congestion, and provides the appropriate responsive actions for the detected incident. Figure 1 shows the system’s framework. The framework is divided into five phases, each representing a step in the overall operation of the intelligent traffic light. The phases of the Passable framework are as follows:

- Phase 1: At every intersection, each road segment is equipped with an intelligent traffic light featuring a CCTV camera and wireless communication technology. The camera, mounted on the traffic light, captures video footage, which is then divided into frames to be sent to the traffic light for processing and analysis. Detection of traffic incidents is performed by an intelligent traffic light controller integrated with two deep learning models, which utilize advanced algorithms, such as YOLO (You Only Look Once). One model is dedicated to detecting accidents, while the other focuses on identifying traffic congestion.
- Phase 2: The intelligent traffic light system utilizes wireless communication to transmit real-time alerts to nearby vehicles whenever a congestion or accident incident is detected. These alerts are tailored to the type of incident, providing drivers with relevant information to make informed decisions, such as rerouting, adjusting their speed, or changing lanes to avoid secondary accidents. By disseminating these alerts, the system enhances road safety by reducing the risk of secondary accidents and improving overall traffic efficiency.
- Phase 3: Upon detecting a road incident, the traffic light dynamically adjusts its cycle to extend green light durations for congested road segments. This adjustment is based on real-time calculations of vehicle density, ensuring smoother traffic flow and reducing congestion.
- Phase 4: All detected incident data is stored in a centralized database. The dashboard interface utilizes this stored information to facilitate real-time monitoring and provide actionable insights for traffic departments.
- Phase 5: Traffic management authorities will use the dashboard interface to monitor traffic flow, adjust traffic control settings, and analyze traffic data to make informed decisions and plan strategically.

## 4. Data Collection and System Requirements

Different data collection methods were employed to gain a comprehensive understanding of the problem from multiple perspectives and identify the requirements of the proposed system. Two primary data-gathering methods were utilized to determine the Passable system requirements: a survey of stakeholders, specifically drivers, and interviews with officers from the General Department of Transportation (GDT). This integrated approach aims to inform and guide the development of the Passable system, ensuring that it effectively meets the needs and expectations of all stakeholders. All participants in the survey or interviews were informed about the system’s objectives, the types of questions, and the anonymous nature of their responses. They were told that participation was voluntary, that they could skip any question or withdraw from the session at any time, and that the collected data would be treated confidentially. Before participating in the survey or interviews, written informed consent was obtained, outlining the data privacy measures and the intended use of the results.

### 4.1. Drivers’ Survey

A comprehensive survey was designed to gather valuable insights from drivers about their experiences with road congestion and accidents, as well as their general attitudes towards the proposed intelligent traffic light system and notification alerts. The survey consisted of 16 questions and was distributed via QR code and social media to reach a broad audience, unrestricted by geography or time zones. Responses were received from 442 individuals. Table 2 shows the demographic information of the participants. The demographic data shows a primarily young population, with 73.6% of participants aged 18 to 24. Geographically, the majority (85.9%) live in the Mecca region, indicating a geographical concentration that may limit overall representativeness in Saudi Arabia. Most of the participants are students (67.4%). Regarding driving habits, a significant proportion (53.4%) do not drive, likely due to their age and student status, whereas 26.3% drive more than five times a week.

Table 3 presents a detailed list of the survey questions, the answer options, and the percentage of responses for each question. A significant majority (90.9%) report suffering from traffic congestion at intersections, highlighting a widespread issue. Notably, 86.5% have experienced waiting at a red light while other directions had no cars, suggesting inefficiencies in traffic light coordination. Furthermore, when asked about the maximum number of times a traffic light cycled before they could pass, 41.6% of respondents said twice, and 31.1% said three times, emphasizing issues with traffic light timing. Additionally, 10.9% of respondents have been involved in a secondary accident due to the occurrence of an accident ahead, while 33.4% know someone who has been involved in such an accident. When asked about accidents caused by sudden acceleration or stops at traffic lights, 20.2% reported having encountered such accidents, while 26.7% know someone who has, and 53.1% have not faced this issue. When estimating wait times at traffic lights, 49.3% of respondents report waiting 5 min or less, while 33.4% wait around 10 min, 11.4% wait around 15 min, and 5.9% wait around 20 min, indicating variability in wait durations. These insights underscore the need for improved traffic management and more efficient traffic light systems to alleviate congestion and reduce wait times. A majority (90%) of respondents prefer to be aware of accidents or severe congestion ahead and change their route accordingly. These results indicate a strong preference for advanced warnings about traffic conditions and highlight the significant impact of traffic accidents and sudden stops at traffic lights on drivers’ safety. When questioned about their willingness to use a vehicle equipped with communication technology for receiving congestion or accident notifications, 82.1% responded affirmatively, 2.1% responded negatively, and 15.8% were uncertain. Furthermore, 64.8% of respondents preferred receiving alerts via voice notifications, 34.9% through text messages, and 63% via light alerts. Additionally, when asked if an AI-powered system would save time, prevent accidents, reduce traffic congestion, and optimize resource use, 80.1% believed it would be helpful, 1.5% did not, and 18.5% were unsure.

### 4.2. Traffic Officer Interviews

Three interviews were conducted to gather information about the current traffic light system. The first interview was unstructured and was conducted with a colonel serving as the Traffic Director of Jeddah City in Saudi Arabia. The second interview was with a colonel serving as the Director of Public Relations. This was undertaken to engage with experts in road safety and traffic control. The third interview, semistructured and consisting of open questions, was conducted with different officers at the GDT. These interviews discussed strategies for managing traffic congestion and mitigating accidents. In addition, the roles of the different departments, including the Accidents Department, Traffic Safety Department, and the Saher Department, were explained.

When asked about the strategies for managing traffic congestion and mitigating accidents, the colonel outlined three primary methods used to identify congested and accident-prone areas: relying on 911 call reports from drivers and traffic police, conducting periodic traffic patrols, and utilizing a network of surveillance cameras. Additionally, when asked about the types of cameras used to detect signal violations, the colonel emphasized the importance of data collection in their operations, highlighting the use of a diverse range of modern and traditional CCTV cameras.

When asked whether they used a current system that is similar to Passable, the colonel discussed a related system called “Sawaher,” which focuses on enhancing security control at Makkah City entrances during Hajj and Ramadan. This system includes individual and vehicle identification and can detect wanted persons traveling through the city. A similar system named “Salim”, currently under review, was also mentioned, aimed at preventing accidents and assisting traffic officers in locating incidents.

The third interview was with the Director of the Traffic Studies Department, which provided insights into the current traffic light system. When asked about the use of modern technology for signal control, the interviewee explained that the current traffic light systems are manually programmed to change signals based on predetermined timings specific to each intersection section. When asked about the primary cause of accidents at intersections, he pointed to sudden stops and changing driving direction as the leading factors.

The series of interviews concluded with valuable insights from an individual who had implemented a similar traffic management system in Tabuk City. This system, similar to Passable but without AI, utilized live cameras on main roads to identify congestion and accidents. This discussion enriched the understanding of practical implementation and offered valuable advice on engaging with relevant authorities to support the system.

### 4.3. Functional and Non-Functional Requirements of Passable System

For the functional requirements, the proposed system should allow authorized users to log in and out, provide administrators with access to traffic statistics and the real-time status of intersections, and record incident details, including congestion and accidents. Additionally, the system should notify drivers of traffic congestion and accidents, respond to real-time incidents by dynamically adjusting traffic signals, and calculate optimal green light durations based on the current traffic conditions.

For non-functional requirements, security is paramount, as it influences traffic management and the safety of people. User authentication and authorization are needed to ensure that only authorized traffic department users can log in using their email and password and access system data.

### 4.4. Data Requirements

The Passable system relies on fundamental data requirements to deliver efficient traffic management and an improved driving experience. These key data elements include incident details such as Incident ID, date, time, image, intersection ID, and type (congestion or accidents). The system tracks the Traffic Light ID, status, number, location, and newly calculated green light time for traffic lights. Intersection data includes Intersection ID, name, area, location, and number of traffic lights within the intersection section. These core data requirements form the basis for Passable’s ability to make real-time decisions and communicate effectively.

### 4.5. Software Requirements and Tools

The software requirements lay the foundation for the development of the Passable system, ensuring that it effectively manages traffic, enhances road safety, and provides an improved driving experience for all users. The software prototype of the Passable system integrates several essential components. The following subsections outline the primary software components of the proposed system, including their key features and requirements.

#### 4.5.1. Traffic Monitoring Dashboard

The designed traffic monitoring dashboard is a user-friendly dashboard interface that allows administrators to oversee the system’s operations, monitor traffic status, and interact with the system. The front end of the monitoring dashboard was designed using Power BI, a powerful business analytics tool developed by Microsoft. It enables users to visualize and analyze data from various sources. With its intuitive interface and robust features, Power BI allows users to create interactive reports and dashboards that can be shared across organizations. Moreover, Power BI seamlessly integrates with Microsoft Azure, facilitating data retrieval. Power BI Desktop version 2.127.1080.0 (https://www.microsoft.com/en-us/power-platform/products/power-bi, accessed on 15 January 2025) is a prominent software program for data visualization and business intelligence. Additionally, Hypertext Markup Language (HTML) and Cascading Style Sheets (CSS) were used to structure and style the contents of the dashboard interface. Moreover, JavaScript was used to create interactive elements and dynamic behavior, enhancing the user experience with the dashboard interface.

For the implementation of the dashboard’s back-end, the Django framework was used. Django provides a high-level abstraction of database operations. It is designed to facilitate the creation of complex, database-driven websites. Django utilizes Python version 3.9 (https://www.python.org, accessed on 15 January 2025) to create scripting code for server-side application logic. Django is a Python web framework renowned for its rapid development capabilities, robust security features, and exceptional scalability. It simplifies web application development with built-in components for database management, authentication, and URL routing. Moreover, it can be integrated with AI models to create web applications with intelligent features. It was used for the front-end and the back-end development to integrate the entire system. It linked the AI models to the database and the AI models’ outputs to the hardware. Visual Studio Code version 1.88 (https://code.visualstudio.com, accessed on 15 January 2025) (VS Code) is a lightweight yet powerful code editor developed by Microsoft. It supports multiple programming languages, offers features such as syntax highlighting and debugging, and features a vast library of extensions for customization. VS Code was used to write the Django system code.

#### 4.5.2. Detected Incidents Database

Microsoft Azure and MySQL were used for database development. Microsoft Azure offers various cloud database services, including Azure Database for MySQL, Azure SQL database, Azure Cosmos DB, and Azure Database for MariaDB. These managed database solutions provide high availability, scalability, and analytics capabilities, enabling businesses to efficiently store and manage their data in the cloud. MySQL is a widely used open-source relational database management system (RDBMS) known for its reliability, scalability, and ease of use. It utilizes Structured Query Language (SQL) for data management and supports various features, including transactions, replication, and user authentication.

#### 4.5.3. AI Models for Accident and Congestion Detection

Two deep learning models are developed to detect accidents and congestion from camera images. For the AI models of the system, the “You Only Look Once” (YOLOv8) algorithm for real-time object detection is utilized, and its performance is compared with the Faster Region Convolutional Neural Networks (Faster RCNN) model. The Yolov8 model was chosen for its speed and accuracy in object detection, making it ideal for real-time traffic analysis. Faster RCNN was selected because it is considered one of the primary models for object detection. The models are implemented in Google Colaboratory (Google Colab), a cloud-based environment for running Python programming language code. Google Colab is a free cloud-based platform provided by Google that allows users to write and execute Python code in a browser-based environment. It provides access to powerful computing resources, including GPUs and TPUs, enabling users to run complex machine learning and data analysis tasks without the need to set up their infrastructure. Additionally, Python is a versatile programming language widely used for web development, data analysis, and Artificial Intelligence (AI). It is known for its simplicity, extensive library, and accessibility to developers of all levels, with strong community support.

#### 4.5.4. Traffic Light Control and Notification Service

Two software modules are needed to control the traffic light operation and notify drivers of detected incidents. One is responsible for calculating and dynamically adjusting the traffic light timings based on real-time traffic conditions and congestion detection. The other software module is responsible for sending real-time alert messages to drivers regarding traffic conditions using wireless communication between the traffic light and the vehicles. Additionally, a software module is responsible for receiving alert messages from vehicles using in-vehicle wireless communication and relaying these messages to other neighboring vehicles.

The Arduino Integrated Development Environment (Arduino IDE) was utilized to develop the traffic light control and notification components. The Arduino IDE is a user-friendly software platform for programming microcontrollers, such as the Arduino and ESP32. It supports the C/C++ programming language. With a simple and intuitive interface, the Arduino IDE enables users to create and control electronic systems by writing code, utilizing multiple libraries, and interacting with sensors, actuators, and other components. It was used to program the ESP32 microcontroller for the prototype.

### 4.6. Hardware Prototype Requirements and Tools

The hardware prototype of Passable consists of several components that work together to optimize traffic light operations and report detected road incidents. The system also enables vehicles to receive alerts and relay them to nearby vehicles.

To facilitate effective operation and testing, several hardware components are required. The system administrator uses a computer system with Internet connectivity to access the Passable dashboard and visualize real-time traffic data. For the traffic light infrastructure, cameras are necessary to monitor traffic conditions in real time. In our prototype, traffic lights are connected to a laptop equipped with a camera and configured to run AI models for detecting road incidents. Additionally, traffic lights must be equipped with wireless communication technologies to send alerts about detected congestion or accidents to nearby vehicles. Similarly, vehicles must have wireless communication capabilities to receive these warnings and exchange information with other nearby vehicles.

**Figure 2:**
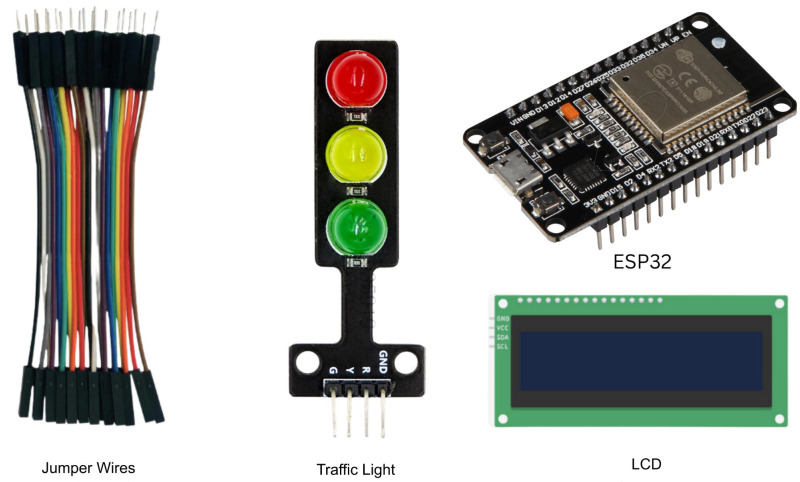
Essential components of hardware prototype.

The core hardware components used in our prototype are illustrated in Figure 2. These include Female-to-Female jumper wires, four 5 V Traffic Light LED Module Boards, a 2 × 16 liquid crystal display (LCD) for showing alert messages to drivers, and an Espressif32 (ESP32) module—a system-on-chip microcontroller with integrated Wi-Fi and dual-mode Bluetooth. Both traffic lights and vehicles are equipped with ESP32 modules, which enable instant transmission and reception of alert messages. Vehicles can also retransmit these alerts to other nearby vehicles.

As a proof of concept, Wi-Fi is used for communication between traffic lights and vehicles, as well as for vehicle-to-vehicle (V2V) communication. The prototype functionality and ESP32 modules are programmed using C++ in the Arduino IDE, a widely used tool for microcontroller development.

In real-world deployments, CCTV cameras are typically installed on traffic lights at intersections. These lights should be integrated with processing units capable of running deep learning models to analyze images and detect incidents. For broader and more robust communication, advanced technologies such as 5G can be used to enhance data exchange between infrastructure and vehicles.

## 5. Passable System Design

The system’s design encompasses the definition of its architecture and modules. It ensures a cohesive understanding of the system’s processes and functions among the development team.

### 5.1. Use Case Diagram

**Figure 3:**
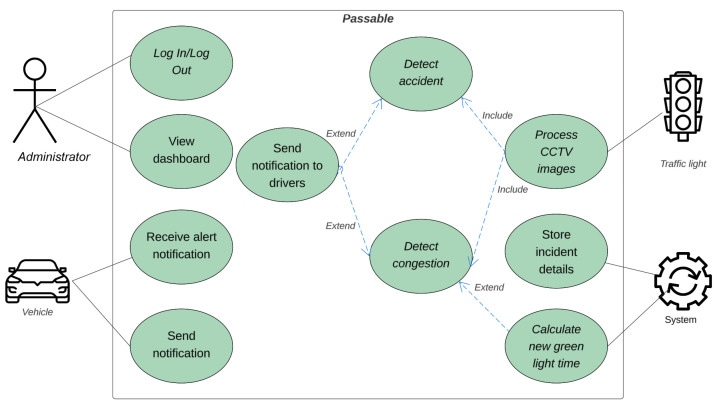
Passable system use case diagram showing actor interactions and core functions, including login, monitoring, AI-based detection, signal adjustment, alerts, and data storage.

The use case diagram for the proposed system, Passable, illustrates the interactions between the different actors (system users). As shown in Figure 3, Passable has four users: the administrator, vehicle, traffic light, and system. The system administrator, who is an officer at the traffic department, has two use cases, which are log in/log out and view statistics. These use cases enable the user to be authorized to access the system and view statistics related to detected congestion and accidents, allowing for the management and oversight of traffic conditions. To authorize users, they must be registered in the database as a system administrator. The traffic light has two main use cases: processing CCTV camera images, a critical function for detecting congestion and accidents, and storing detected incident details on the server. The “detect congestion” and “detect accident” use cases are central to the system, both of which include the processing of CCTV images. These detection use cases extend actions like changing the green light timer and sending notifications to drivers, ensuring adaptive responses to traffic conditions and incidents. Based on the detected accidents or congestion incidents, the green traffic light timer is updated and an alert message is sent to the drivers using in-vehicle wireless communication. Additionally, the incident details are stored in the database.

### 5.2. Passable System Flowchart

**Figure 4:**
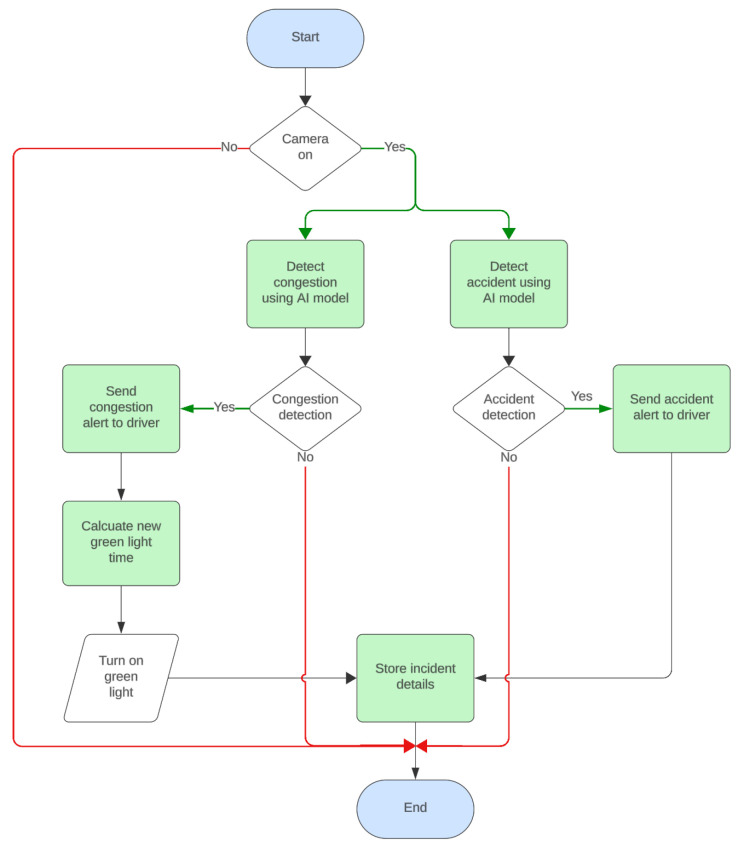
Passable system flowchart from camera activation to concurrent accident and congestion detection using dedicated AI models, adaptive signal timing, driver alerts, and data storage.

Figure 4 illustrates the logical flow of actions and decision-making steps within the system, which is essential for demonstrating efficient traffic management using the proposed system, Passable. The flowchart outlines the operation of the traffic management system, which utilizes AI models to monitor road conditions and detect congestion and accidents. This approach ensures real-time response and adaptive traffic management based on current road conditions. The process starts when the camera is turned on. If the camera is off, the system loops back to the start. When the camera is on, it simultaneously checks for congestion and accidents using two AI models. If congestion is detected, an alert is sent to the driver, followed by the calculation of a new green light time to alleviate traffic. The green light is then activated, and incident details are stored. If no congestion is detected, the system loops back to continuously monitor conditions. Similarly, if an accident is detected, an alert is sent to the driver, and incident details are stored. If no accident is detected, the system loops back for continuous monitoring. The process ends after storing the incident details.

### 5.3. Sequence Diagrams

**Figure 5:**
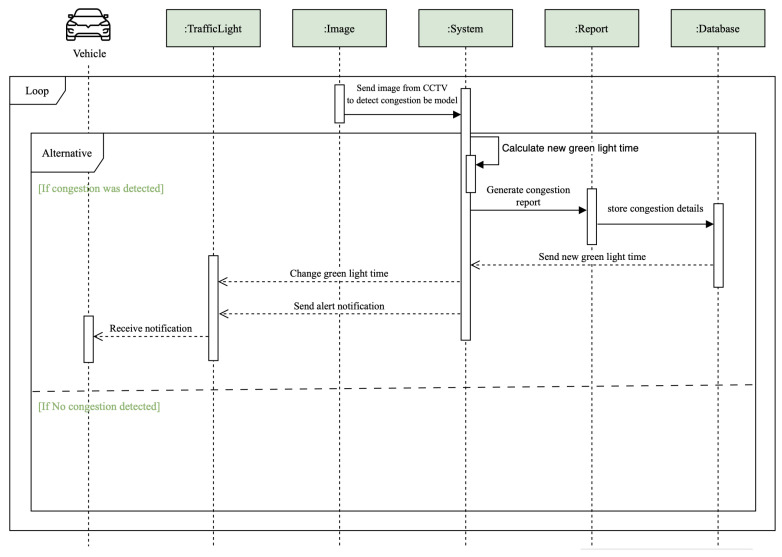
Sequence diagram for congestion detection.

Sequence diagrams illustrate the interactions between the system’s entities and the output of each scenario. Figure 5 represents a sequence diagram for congestion detection, which outlines the interactions between various system components when traffic congestion is detected. The process begins in a continuous monitoring loop involving a vehicle and a traffic light. The traffic light captures an image from CCTV and sends it to the system for analysis. The system processes the image to detect congestion using a deep learning model. If congestion is detected, the system calculates a new green light time to alleviate the congestion. A congestion report is generated and stored in the database. Concurrently, the new green light time is sent to the traffic light to adjust its timing, and an alert notification is sent to the vehicle to inform the driver of the congestion. If no congestion is detected, the process loops back and continues monitoring. This sequence ensures that the system dynamically adjusts traffic light timings and informs drivers instantly, enabling them to manage and mitigate congestion effectively.

**Figure 6:**
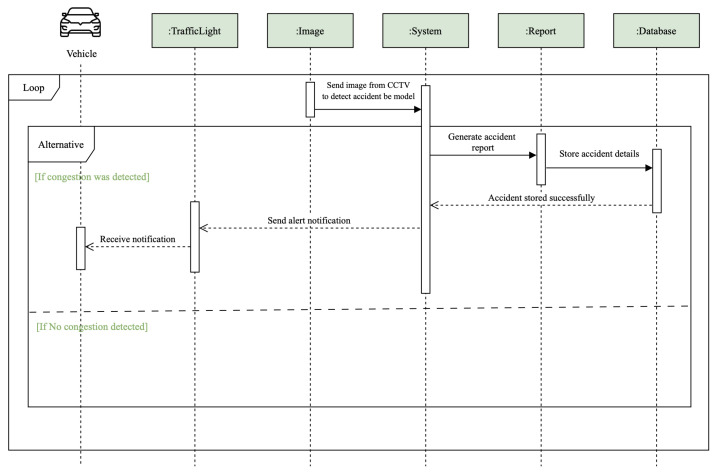
Sequence diagram for accident detection.

Figure 6 presents the sequence diagram for accident detection. The sequence diagram for accident detection outlines the interactions among different components of the traffic management system when an accident is detected. The process begins with the continuous monitoring loop involving a vehicle and a traffic light. When an image from a CCTV camera is captured, it is sent to the system for analysis. The system uses a deep learning model to detect accidents from the image data. If an accident is detected, the system generates an accident report. This report is then sent to the database, where accident details are stored, confirming that the accident has been successfully logged. Concurrently, an alert notification is sent to the vehicle, informing the driver of the accident. If no accident is detected, the loop continues to monitor without issuing any notifications or reports. This sequence enables the system to efficiently detect and respond to traffic accidents, thereby helping to manage traffic flow and inform drivers in real time.

**Figure 7:**
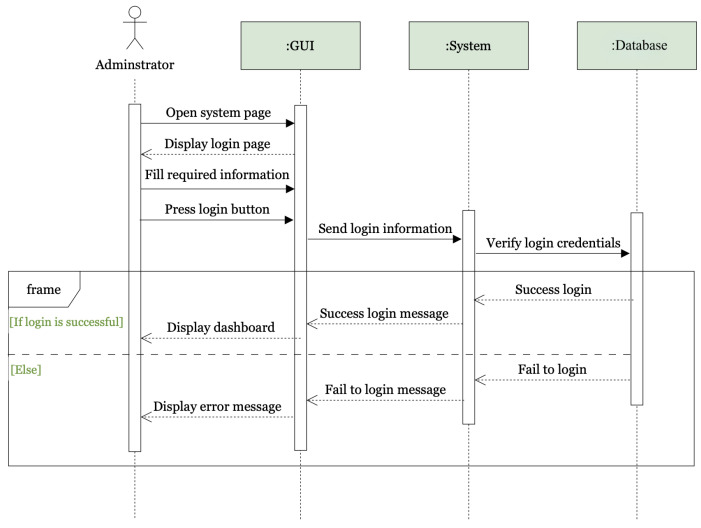
Sequence diagram for dashboard login.

Figure 7 illustrates the sequence for administrators’ logins, outlining the flow of information and actions taken to authenticate administrators to the dashboard. The sequence diagram for the login process illustrates the steps involved when an administrator logs into the system. The administrator starts by opening the system page through the GUI, which then displays the login page. The administrator fills in the required login information and presses the login button. This action sends the login information to the system, which in turn verifies the login credentials by querying the database. If the credentials are verified successfully, the database sends a success message back to the system, which then forwards this success message to the GUI. Consequently, the GUI displays the dashboard to the administrator, indicating a successful login. If the credentials are not verified, the database sends a failure message to the system, which then forwards this failure message to the GUI. The GUI subsequently displays an error message to the administrator, indicating that the login attempt was unsuccessful.

### 5.4. Traffic Light Control Algorithm

**Figure 8:**
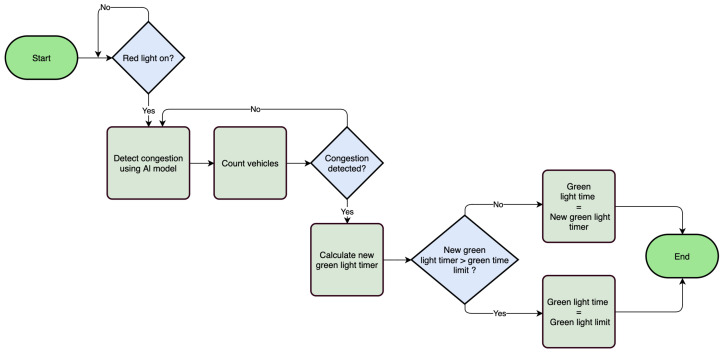
Flowchart of AI-based traffic light control, showing vehicle counting, green time calculation, and decision points for signal timing limits.

The proposed system is designed to efficiently manage traffic flow by operating traffic signals based on the density of vehicles present on the road. The AI model used for congestion detection counts the number of vehicles in the current lane to calculate the appropriate time for the green light based on the detected density. The flowchart, shown in Figure 8, illustrates a dynamic traffic light control system that utilizes AI to optimize green light duration based on real-time traffic conditions. The process begins by checking if the red light is currently on. If so, an AI model is activated to detect traffic congestion and count the number of vehicles present. If congestion is detected, the system calculates a new green light timer based on the traffic volume. This newly calculated timer is then compared to a predefined maximum green light limit. If the computed duration exceeds the limit, the green light time is set to the maximum allowed value; otherwise, it is updated to the new calculated duration. If no congestion is detected or if the red light is not active, the system concludes without modifying the green light duration.

The traffic light switching algorithm is responsible for dynamically adjusting the duration of green signals based on the detected vehicle count. The algorithm steps are presented in Algorithm 1. The algorithm is implemented in Django using Python. It is used for calculating the signal time for the hardware prototype. The algorithm takes the number of lanes and the minimum, maximum, and default green light timings as input. Additionally, the vehicle count is accepted as input from the detection module. During the initial execution, the green light timing is set to the default timer. Subsequent signal green light timings are calculated using the following equation adopted from [37]:

(1) $$GLT=\left\lceil \frac{\sum_{i=1}^{N_{vehClass}}\left(vehCount_{i}\times D_{h_{i}}\right)}{\;numLanes+1}\right\rceil$$

where GLT is the green light time, vehClass denotes the vehicle class or type, and $vehCount_{i}$ represents the number of detected vehicles of a specific class. The $D_{h_{i}}$ is the average time gap between consecutive vehicles crossing an intersection stop line during the green light phase. It varies according to vehicle type, intersection distance, and traffic conditions  [38]. The numLanes is the number of lanes in the road section. Although Equation (1) is general enough to incorporate multiple vehicle classes (e.g., cars, trucks, bicycles), only cars were considered in our implementation. Additionally, bicycles and other non-motorized vehicles were excluded due to their minimal impact on intersection-level congestion. The $D_{h_{i}}$ for cars is approximately equal to 2 s under normal traffic conditions. Assuming steady traffic flow and uniform vehicle behavior, $D_{h_{i}}$ was set at 2.6 s in this study to account for start-up delays and slower traffic flow.
**Algorithm 1** Green light time calculation.  1:**function** GLT(vehCount)  2:      **if** initial execution **then**  3:            GLT←default  4:      **else**  5:            $D_{h}\leftarrow 2.6$                                                                  ▹ Define parameters  6:            numLanes←2  7:            minDefault←20  8:            maxDefault←40  9:            $GLT\leftarrow \lceil \frac{vehCount\times D_{h}}{numLanes+1}\rceil$                                                ▹ Calculate GLT10:            **if** GLT<minDefault **then**            ▹ Ensure GLT within allowed range11:                 GLT←minDefault12:            **else if** GLT>maxDefault **then**13:                 GLT←maxDefault14:            **end if**15:      **end if**16:      **return** GLT17:**end function**

## 6. Passable Deep Learning Models

In this section, the dataset and the development of the deep learning models used in Passable are discussed.

### 6.1. Passable Images Dataset

Passable utilizes images captured by CCTV surveillance cameras installed at intersections. These cameras record video footage, which needs to be divided into images (frames) for analysis. The images should be colored to ensure a more realistic training. Additionally, the images should have a minimum resolution of 720p, allowing the deep learning model to identify patterns and features more accurately. The angle of the CCTV camera affects the model’s functionality. In general, three angle types are used in AI models for detecting objects: eye-level, high-angle, and overhead views. CCTV cameras usually use high-angle images because they provide a good overview of open areas, such as streets and intersections.

For the congestion detection task, the Traffic Object Detection Dataset was chosen, incorporating two versions to form a comprehensive dataset. The first version (https://universe.roboflow.com/inlights-s5jdz/traffic-sfpps/dataset/5, accessed on 20 May 2025) contained 1261 images, while the second version (https://universe.roboflow.com/inlights-s5jdz/traffic-sfpps/dataset/1, accessed on 20 May 2025) included 154 images, with grayscale applied to 25% of them. By combining these two versions, a dataset of 1415 images was created. For the accident detection task, the Car Crash Detection Image Dataset (https://universe.roboflow.com/mada-study/car-crash-detection-c9xlj/dataset/1, accessed on 20 May 2025), containing 905 images, was selected. The datasets were chosen because they included critical factors such as lighting and distance variations, ensuring the model could detect congestion or accidents both at night and from various distances.

### 6.2. Building the Deep Learning Model

Two YOLOv8 models were trained using the selected datasets of congestion and accidents, with both datasets divided into 70% for training, 20% for validation, and 10% for testing. For the congestion detection dataset, the model was trained on a single class, which is “Car,” and for the accident dataset the model was trained on one class, which is “Crash.”

### 6.3. Model Configuration and Training

Six experiments were conducted to obtain the final congestion detection model with the best performance. Different hyper-parameters were tested in each of the six experiments to determine the optimal performance. In the first experiment, the model was trained with an epoch number of 150 and a batch size of 16. The number of epochs was then gradually increased to 250, with increments of 50 in each subsequent experiment. For each epoch number, two different batch sizes, 16 and 32, were used. The metrics used to evaluate the model’s performance on the training dataset included accuracy, mAP50 (Mean Average Precision), precision, and recall. After the best hyper-parameters were identified, they were used for model validation and testing. A summary of the experiments conducted for the congestion detection model is provided in Table 4. It is evident that there is no significant difference between the experiments. The hyper-parameter values from the second experiment, which involved a batch size of 32 and 150 epochs, were selected due to their higher accuracy and mAP values compared to those from the other experiments.

Six experiments were conducted to develop the final accident detection model with optimal performance. In each of the six experiments, hyper-parameters were varied, starting with a batch size of 16 and training for 150 epochs. The number of epochs was then gradually increased to 250, with increments of 50 epochs in each subsequent experiment. Two different batch sizes, 16 and 32, were used in each experiment. The training experiments for the accident detection model are summarized in Table 5. As indicated in the table, the hyper-parameter values from the second experiment, with a batch size of 32 and 150 epochs, produced the best results, as these settings achieved the highest accuracy and mAP values among all the experiments.

### 6.4. Model Testing and Evaluation

Once the models had been trained on the congestion and accident training sets, the best hyper-parameters, which were found to produce the highest accuracy, were selected. As shown in Table 4 and Table 5, these hyper-parameters were 150 epochs and a batch size of 32 for both models. The best weights identified for the models were then loaded and used for validation and testing. The validation set was used to validate the model, and the model was subsequently tested on the test dataset, as shown in Table 6.

**Figure 9:**
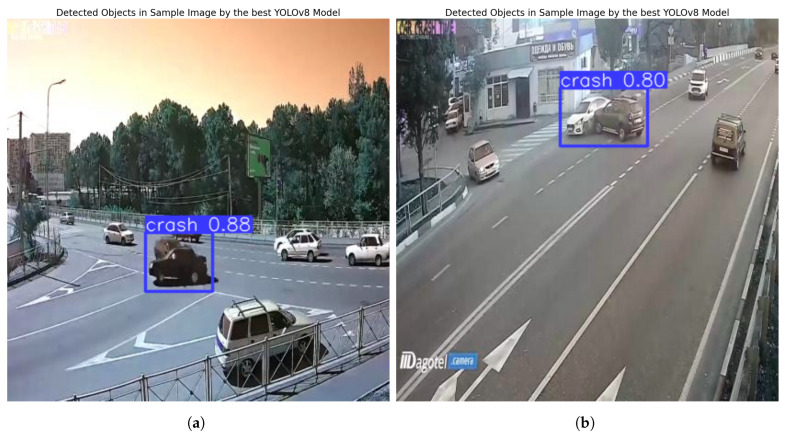
Sample accident detection results with annotated bounding boxes and different confidence scores of (**a**) 0.88 and (**b**) 0.80 for the detected accident regions using YOLOv8.

**Figure 10:**
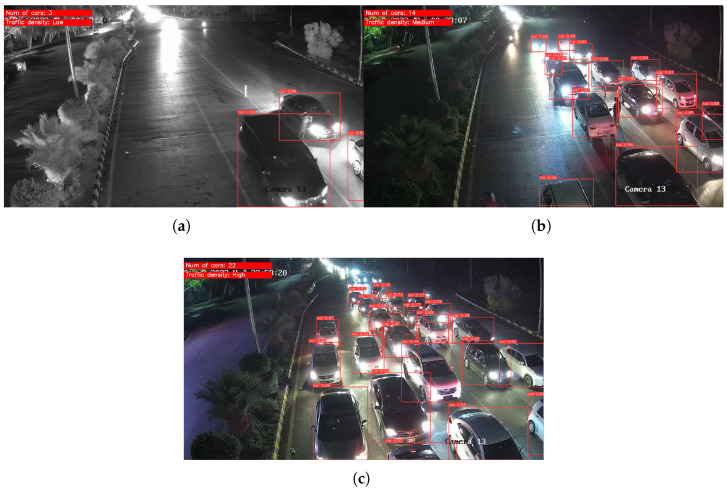
Sample congestion detection results showing annotated vehicle bounding boxes and different levels of (**a**) low, (**b**) medium, and (**c**) high density classifications generated by the YOLOv8 model.

Figure 9 shows sample accident images after testing. Figure 10 illustrates the three traffic density levels in the congestion dataset tests. Traffic density is classified based on the number of detected cars, as shown in Table 7. Motorbikes are excluded, as they do not significantly affect congestion [39].

## 7. Passable System Implementation

This section focuses on the implementation of Passable, highlighting the employed tools and technologies, the calculation of green signal time, the deployment of the dashboard interface, and hardware implementation.

### 7.1. Dashboard Implementation

**Figure 11:**
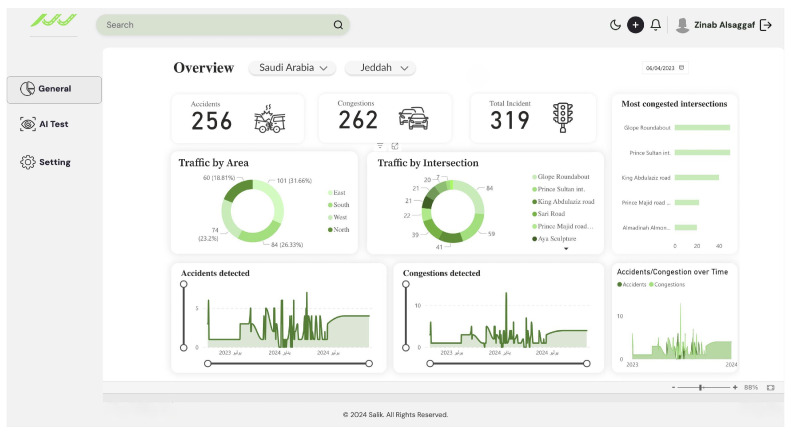
Dashboard visualizing traffic conditions and displaying congestion and accident statistics.

The dashboard web page, shown in Figure 11, visualizes real-time traffic conditions and displays congestion and accident statistics. It was built with Power BI and integrated with Microsoft Azure. This integration ensures the dashboard remains connected to the underlying database, automatically updating as synchronization occurs to provide the most recent information.

The dashboard contains three tabs on the left. The General tab, which is the main page, contains an overview of incident statistics. The AI Test tab allows users to upload an image and test the AI models. Lastly, the Settings tab is for general settings. The General tab of the dashboard has nine sections. The top of the General tab page includes three sections, which display the number of detected accidents, congestion incidents, and total number of road incidents. The second part of the General tab displays traffic distribution by city area and street name. The section of the most congested intersections displays the most congested intersections in the city. The bottom of the dashboard interface displays the accidents and congestion incidents detected in the city using a time frame, which provides a visualization of the changes in the number of incidents detected over time. The dashboard changes dynamically based on the user’s input on one of the section’s icons. For example, if the user selects the “Traffic by area” section, all other sections will update based on the chosen area.

### 7.2. Hardware Implementation

**Figure 12:**
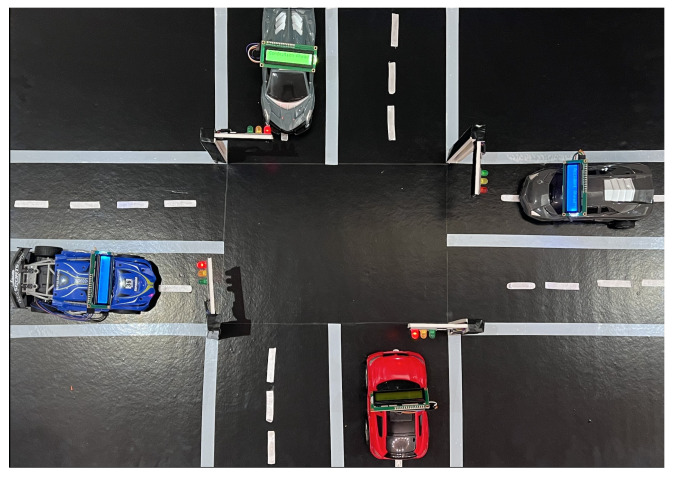
Passable system hardware prototype.

To demonstrate the functionality of our system, we created a hardware prototype that depends primarily on the ESP32 microcontroller. The prototype, shown in Figure 12, includes two main components: the traffic light and vehicle components. The traffic light adjusts the green light timing based on detected congestion, thereby improving traffic flow, and sends alerts to vehicles. The vehicle component shows how vehicles receive alert messages about traffic conditions.

**Figure 13:**
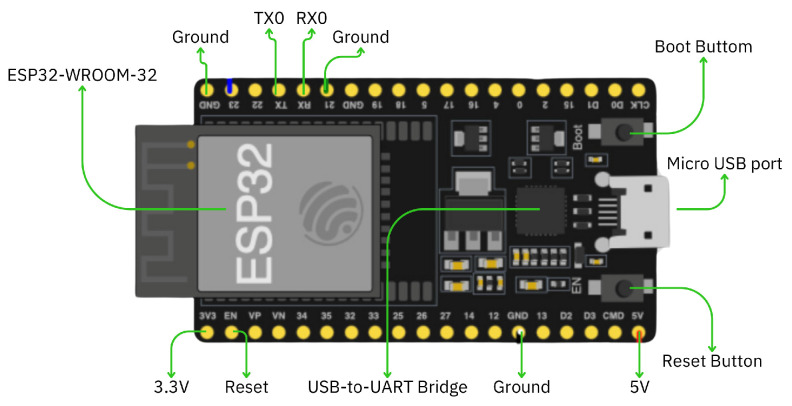
ESP32 microcontroller parts.

Traffic lights and vehicles are equipped with the ESP32 microcontroller, a versatile board for IoT and embedded applications. One notable feature of the ESP32 is its support for Wi-Fi and Bluetooth connectivity, enabling wireless communication capabilities. Moreover, the module offers compatibility with various communication protocols and can be programmed using widely used development platforms such as Arduino IDE and Visual Studio. The different parts of the ESP32 microcontroller are illustrated in Figure 13. The ESP32 microcontroller board includes the ESP32-WROOM-32 module for Wi-Fi and Bluetooth communication. Key pins include 3.3 V and 5 V for power, GND for ground, and TX0/RX0 for serial communication. It has a Reset Button for rebooting and a Boot Button for entering firmware mode. The USB-to-UART Bridge enables USB communication via the Micro USB port for programming and power. Additional pins support external connections, while the Reset Pin allows hardware resets. The ESP32’s ability to send and receive data using Wi-Fi was utilized in the proposed hardware prototype.

**Figure 14:**
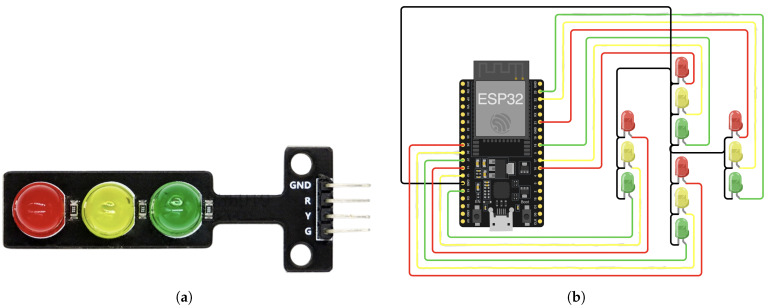
Hardware components of the traffic light system, including (**a**) LED signal modules, and (**b**) wiring connections and the ESP32 microcontroller used for control and communication.

Each traffic light module, shown in Figure 14a, includes four pinouts, with GND representing ground, G for the green light, Y for the yellow light, and R for the red light. The ESP32 microcontroller is responsible for controlling the four traffic lights and transmitting messages to vehicles. The software developed to control the traffic light at the intersection was downloaded to the ESP32 microcontroller. To address the limited number of ground (GND) pins on the ESP32, the existing GND pins, which serve as a zero-voltage reference point, were extended. This was achieved by connecting five Female-to-Female wires together, effectively creating a shared ground connection for multiple components. This adjustment ensures a stable common ground for the circuit, preventing potential voltage inconsistencies across connected devices.

**Figure 15:**
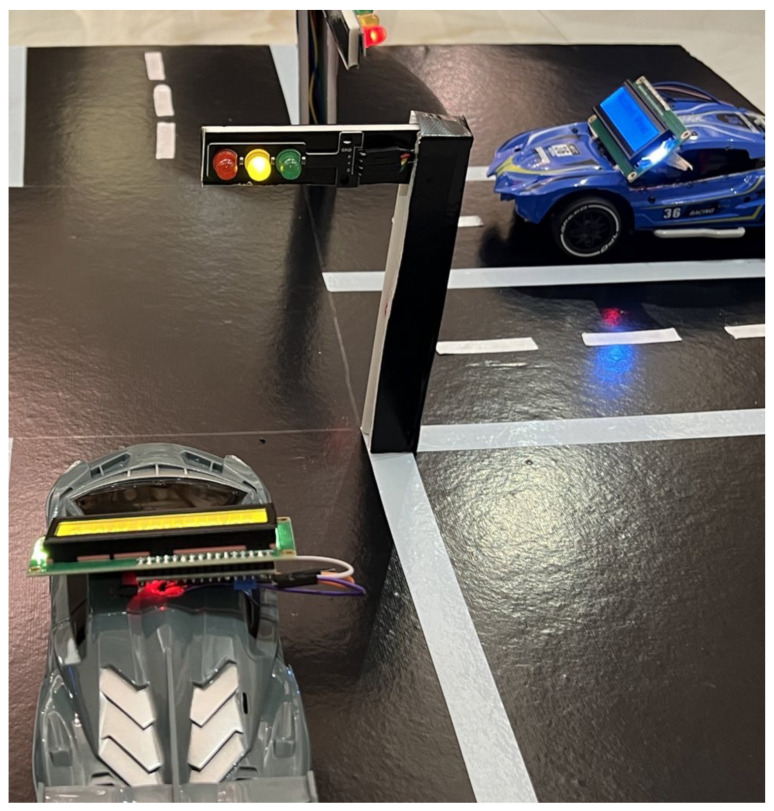
The traffic light at the intersection prototype model.

Figure 14b depicts the final circuit configuration for the traffic light. One wire was connected to the ground pin at the ESP32 microcontroller, which is shown in black color, while the remaining four wires were allocated to each individual traffic light module. The diagram illustrates the wiring of traffic light modules to the ESP32 microcontroller, where the wire colors correspond to the connections of each LED (green, yellow, and red) within the traffic light modules. Traffic Light 1 is connected to the ESP32 as follows: the green LED to pin 23, the yellow LED to pin 22, and the red LED to pin 21. For Traffic Light 2, the green LED connects to pin 17, the yellow LED to pin 18, and the red LED to pin 19. Traffic Light 3 has its green LED connected to pin 25, the yellow LED to pin 26, and the red LED to pin 27. Finally, Traffic Light 4 connects the green LED to pin 14, the yellow LED to pin 16, and the red LED to pin 13. The black wires in the diagram represent the shared ground (GND) connections, ensuring a common zero-voltage reference for all LEDs. Figure 15 shows the traffic light operation after connecting it to the microcontroller and installing it at the intersection prototype.

**Figure 16:**
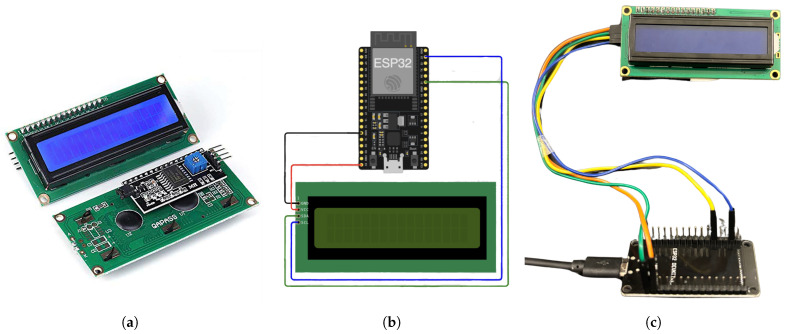
Vehicle-side hardware showing (**a**) the alert display module, (**b**) its wiring schematic, and (**c**) the physical implementation within the prototype.

One of the important features of our proposed system, Passable, is the wireless communication capability that enables vehicles to be alerted to detected road incidents. To demonstrate this functionality in the prototype, an ESP32 microcontroller was utilized for each vehicle to receive data transmitted by the traffic light. Four vehicles were used in the proposed prototype, one at each traffic light. A 16 × 2 liquid crystal display (LCD) with an Inter-Integrated Circuit (I2C) module interface was used to simplify wiring and reduce the number of required GPIO pins on the ESP32. The LCD with I2C are shown in Figure 16a. The I2C interface allows the LCD to communicate with the microcontroller using only two pins, SDA (data line) and SCL (clock line), enabling efficient integration while maintaining a clear and readable text display. Figure 16b illustrates the vehicle’s circuit connections, while Figure 16c shows its implementation.

**Figure 17:**
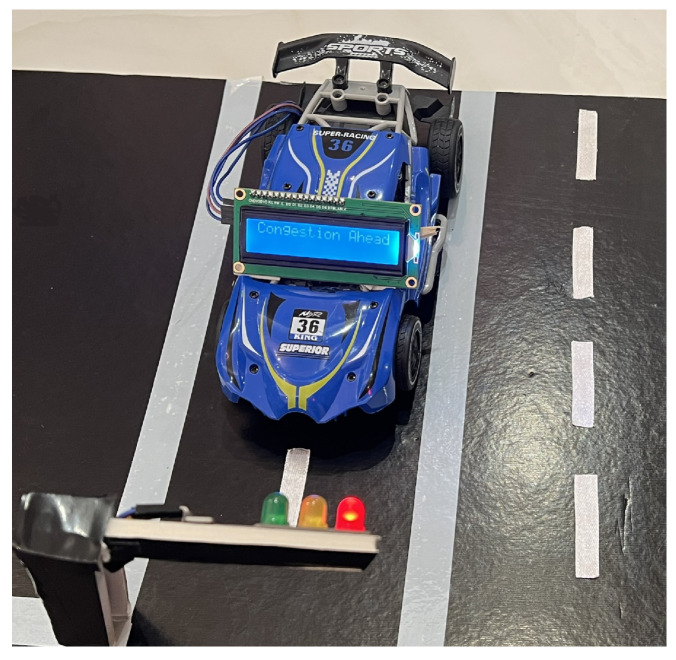
LCD displays congestion alert message received from the traffic light.

To enable data reception, the MAC address of each ESP32 microcontroller was retrieved, stored, and then integrated into the traffic light code. In addition, the LCD display was identified to allow printing of relevant information. Upon powering up, the vehicles receive alerts whenever they are sent, as shown in Figure 17.

Integrating the ESP32 microcontroller with the Django web application for processing the AI models and calculating GLT involves setting up communication between the ESP32 and the Django server using HTTP requests. The ESP32 sends an HTTP GET request to the Django server to transmit traffic information data. A Django view is configured to receive and process these GET requests on the server side. The view detects if an accident occurs; detects the traffic density and determines whether it is low, medium, or high and calculates the new green light time; and sends back a JSON response to the hardware. An example of the JSON response follows:


     {
	    "is_accident": false,
	    "is_congestion": true,
	    "new_green_light_time": 30
     }


## 8. Passable System Testing and Evaluation

System testing is an essential phase of system development that verifies the system’s performance and functionality. Various testing techniques were employed throughout the development of Passable to ensure the system’s consistent performance and functionality. The testing types include unit testing, back-end testing, integration testing, and usability testing. Additionally, hardware testing was performed to ensure proper functioning and effectiveness of the hardware components.

### 8.1. Unit Testing

The unit testing includes testing the dashboard interface and the AI model interface. The dashboard’s unit testing focuses on validating the functionality of individual pages, as well as the behavior and responsiveness of each page’s features. The results confirmed that all components operated as intended, with no major detected defects.

**Figure 18:**
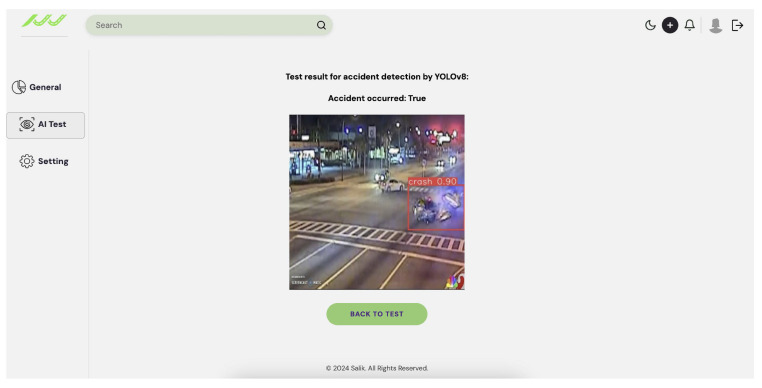
Accident detection test page results using the YOLOv8 model with annotated bounding boxes and confidence scores.

**Figure 19:**
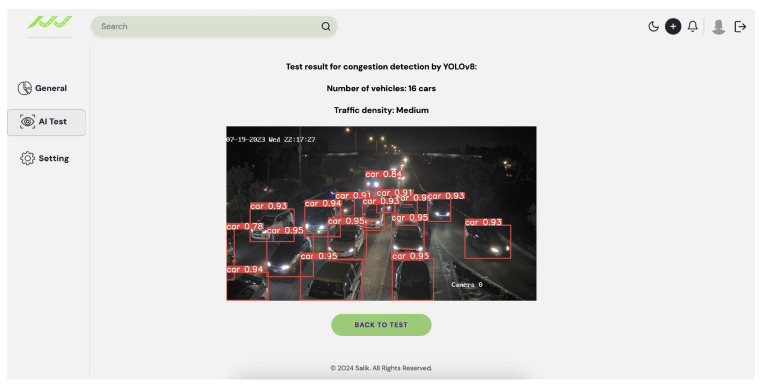
Congestion detection test page results using the YOLOv8 model with annotated vehicle bounding boxes and traffic density classification.

To evaluate the performance of the developed AI models for congestion and accident detection, a dedicated test page was designed and integrated with the models using Django. The testing process utilized new, previously unseen images to ensure unbiased evaluation. The interface allows users to upload an image and select either accident or congestion detection. The models accurately detected all relevant objects, successfully identifying congestion and accidents with high accuracy. As illustrated in Figure 18, the output for an accident image consists of a Boolean result indicating the presence of an accident, accompanied by an annotated image highlighting the detected event. The output for a congestion image, as shown in Figure 19, includes the estimated congestion density, the number of detected vehicles, and an annotated image highlighting the identified vehicles.

### 8.2. Hardware Prototype Testing

Hardware testing is essential to ensure the proper functioning of system components. We conducted tests to evaluate the interaction between the hardware prototype elements, with a particular focus on wireless connectivity using Wi-Fi. The primary objective was to verify the wireless communication between the traffic light and vehicle modules by determining whether the ESP32 microcontrollers in the vehicles could successfully receive data from the traffic light’s ESP32 module.

**Figure 20:**
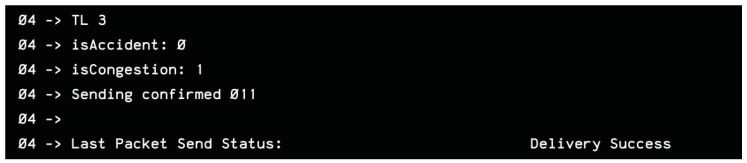
Status of delivering the alert shown on the serial monitor.

To assess connection success, we initially relied on the LCD display to present the data received via Wi-Fi. However, the display occasionally failed to show information, either due to connection issues or display-related errors. To facilitate debugging, we implemented a terminal-like interface using the serial monitor, where the message delivery status was printed. If the vehicle was not detected on the network, the message “Delivery Fail” was displayed; otherwise, a successful transmission was indicated by “Delivery Success”, as shown in Figure 20.

**Figure 21:**
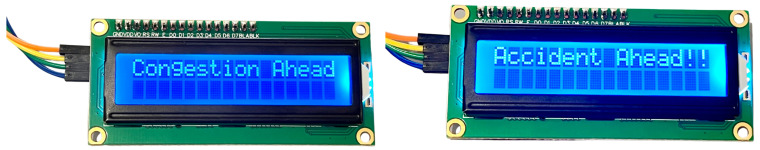
Display alert message on vehicles’ LCDs.

Additionally, we tested the responsiveness of the vehicle-mounted LCDs to alert messages received from the traffic lights. To simulate various detection scenarios for congestion and accidents, we assigned random binary values—where 1 indicated the detection of an event (accident or congestion) and 0 indicated no detection. These values were printed on the serial monitor for verification, while corresponding alert messages were displayed on the LCD screen, as illustrated in Figure 21.

Another crucial aspect of hardware testing was the integration testing of all hardware components. The objective was to ensure that the intersection was correctly controlled and that all traffic lights operated in the correct sequence and order. The testing produced positive results, confirming that the intersection hardware functioned as intended and that all traffic lights performed correctly.

### 8.3. Integration Testing

Integration testing ensures the whole work of the system’s components. Any action taken at the inputs will affect the outputs accordingly. For the dashboard–database integration testing, when adding any entry to the tables connected to the dashboard (e.g., an accident is detected), the dashboard will automatically be updated, and the numbers will be incremented.

**Figure 22:**
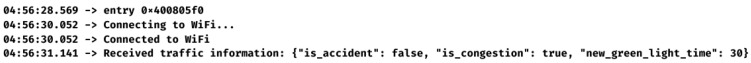
JSON response from Django server.

During the hardware–Django server integration testing, the response to an HTTP GET request was successfully received and displayed in the Arduino IDE serial monitor, as illustrated in Figure 22, confirming proper integration.

### 8.4. Dashboard Usability Testing

Usability testing is a method used to evaluate the effectiveness and user-friendliness of a user interface (UI). For our system, Passable, the objective of usability testing was to assess how intuitive and easy the dashboard interfaces are for GDT users, and how well these interfaces support them in achieving their tasks. The process involved setting up a controlled testing environment, selecting appropriate participants, and observing their performance as they completed specific usability tasks.

Five GDT employees with experience in managing traffic-related issues participated in the dashboard testing, including four traffic officers and one traffic manager. Participants’ ages ranged from 28 to 45 years. The usability test was conducted at the GDT office in Jeddah, in a semi-quiet environment that closely simulated their actual workplace conditions. Participants were asked to complete eight core tasks that covered key dashboard functionalities. The test followed an unmoderated format, allowing participants to perform the tasks independently without real-time guidance.

Participants were briefed and informed about the testing’s objectives, the specific tasks they would be expected to perform, and how their interactions with the system would be observed to evaluate the system’s usability. They were informed that no personally identifiable information would be stored on file and that the sole purpose of their feedback would be to assess and improve the system’s usability. Before participation, which was entirely voluntary, written informed permission was acquired. The consent form contained details regarding participant rights, data processing, and the non-invasive nature of the testing procedure.

Participants were evaluated based on the time it took to complete tasks and the number of clicks required. In addition, they rated the ease of each task and their overall satisfaction with the dashboard experience. Table 8 presents the usability tasks assigned to participants along with the corresponding success criteria, defined by completion time (in seconds) and number of clicks. The participants’ performance was then analyzed in relation to these criteria to assess task efficiency and effectiveness.

All participants successfully completed the tasks. The time taken to complete each assigned task is recorded for each user in seconds. Table 9 shows the task completion time for each user and the average completion time per task. The task completion time results indicate that most dashboard functions were performed efficiently, with tasks such as retrieving traffic data (Tasks 3, 5, 7, and 8) completed in under 5.5 s on average, reflecting good usability and intuitive design. Moderately time-consuming tasks like login and regional queries (Tasks 2 and 6) suggest minor interaction complexity but remained within acceptable limits. Task 1, involving user sign-up, had the highest average completion time (33.2 s), indicating a potential need to simplify the registration process. Task 4 also showed higher variability, suggesting that region-based filtering could benefit from interface improvements. Overall, the results demonstrate a satisfactory level of efficiency, with opportunities for refinement in a few areas.

The number-of-clicks metric measures the total number of clicks performed by users to complete a task. Table 10 shows the number of clicks performed by each user to accomplish each of the assigned tasks and the average number of clicks per task. The number of clicks required to complete each task provides insight into the efficiency and simplicity of the dashboard interface. Tasks 3 and 8 had the lowest average number of clicks (0 and 1.2, respectively), indicating that these actions were either automated or accessible through a single view. Similarly, Tasks 2, 5, and 7 required minimal interaction, averaging three clicks or fewer, thereby suggesting a straightforward user flow. In contrast, Task 1 (sign-up) involved the highest number of clicks (an average of six), reflecting its more complex nature, possibly due to form field requirements. Tasks 4 and 6 showed the most variability and the highest interaction level among data retrieval tasks (an average of 3.4 and 3.8 clicks, respectively), indicating potential room for streamlining the steps involved. Overall, the click data confirms that most tasks were completed with minimal effort, supporting the dashboard’s usability goals.

After completing all the tasks, participants were asked to rate the difficulty of each task as easy, moderate, or hard. The results showed that four out of five participants (80%) rated the dashboard as easy to use, while one participant (20%) reported a moderate level of ease. Participants were also asked about their overall satisfaction with the dashboard. All participants (100%) indicated that they were satisfied with their experience. Overall, the dashboard was well-received by participants for its clean and modern interface.

Participant feedback provided valuable insights into usability and areas for enhancement. Common suggestions included adding more detailed sections, integrating a map to visualize incident locations, and offering personalization and customization features. While some users reported smooth and responsive interactions, others experienced occasional lag or slow loading times, indicating potential areas for performance optimization.

### 8.5. Analytical Performance Evaluation

To illustrate the effectiveness of the proposed adaptive traffic light system, an analytical performance evaluation was performed based on the Highway Capacity Manual (HCM 6th Edition) [40]. Similar analytical approaches have been used in adaptive signal control research to quantify improvements in intersection performance without full-scale field testing, such as in [13,16,41,42]. The analysis considered a conventional fixed-time traffic signal with a cycle duration of 60 s, of which 20 s were allocated to the green phase. In the proposed adaptive approach, the green light time GLT is calculated using Equation (1). In this study, only the car vehicle class is considered, with an average vehicle passing time of 2.6 s. Thus, the equation can be written as

(2) $$GLT_{cars}=\left\lceil \frac{vehCount\cdot 2.6}{numLanes+1}\right\rceil$$

The capacity per cycle can be defined as the maximum number of vehicles that can pass through an intersection during the green phase of a single signal cycle. Given the number of lanes *L*, per-lane saturation flow rate $S_{R}$, and green phase duration GLT, the number of served vehicles in each signal cycle can be calculated using the following equation:

(3) $$Capacity_{cycle}=L\cdot S_{R}\cdot GLT\;\left[\mathrm{veh}/\mathrm{cycle}\right],$$

With an average discharge headway $D_{h}$ of 2.6 s per vehicle, the per-lane saturation flow rate can be estimated as $S_{R}=\frac{1}{D_{h}}=0.385$ vehicles per second. Assuming a scenario with two lanes, multiplying $S_{R}$ by two yields a total discharge rate $D_{t}$ of 0.7692 vehicles per second. Therefore, the total number of vehicles served during the green phase (capacity per cycle) for the two lanes can be written as

(4) $$Capacity_{cycle_{2L}}=0.7692\cdot GLT\;\left[\mathrm{veh}/\mathrm{cycle}\right],$$

For example, with GLT=20 s, the capacity is approximately 15.38 vehicles per cycle, and with GLT=38 s, the capacity increases to 29.23 vehicles per cycle.

To evaluate the effectiveness of the proposed adaptive traffic light approach, the average delay and traffic throughput are used as evaluation metrics. Assuming uniform (non-random) arrivals and no initial queue, the average Delay per vehicle for an undersaturated signal can be approximated as

(5) $$Delay\approx \frac{RLT^{2}}{2CL}\;\left[s/\mathrm{veh}\right],$$

where CL is the cycle length in seconds and RLT=CL−GLT is the effective red time in seconds. The delay approximation in Equation (5) is based on Webster’s classical uniform delay model [40,43], and it is consistent with its simplified treatment in [44].

Traffic throughput TP is the actual number of vehicles that have passed through an intersection in a given period of time and can be calculated using the following equation:

(6) $$TP=\min\;\left(Demand,\;\frac{Capacity_{cycle}}{CL}\times 60\right)\;\left[\mathrm{veh}/\min\right].$$

where Demand is the traffic arrival rate measured by the number of vehicles per unit time.

The percentage reduction in average delay is calculated as

(7) $$DelayReduction\left(\%\right)=\frac{Delay_{fixed}-Delay_{adaptive}}{Delay_{\mathrm{fixed}}}\times 100\%.$$

where $Delay_{fixed}$ and $Delay_{adaptive}$ are the average delays of the fixed and adaptive traffic lights, respectively, calculated using Equation (5).

Similarly, the percentage of throughput improvement is

(8) $$TP\left(\%\right)=\frac{TP_{adaptive}-TP_{fixed}}{TP_{fixed}}\times 100\%.$$

where $TP_{adaptive}$ and $TP_{fixed}$ are the traffic throughput of the adaptive and fixed traffic lights, respectively, calculated using Equation (8).

To evaluate the effectiveness of the proposed system, three traffic density scenarios consistent with the density classification in Table 7 were considered: low density (6 vehicles per minute), medium density (18 vehicles per minute), and high density (28 vehicles per minute). To ensure comparability, the evaluation was benchmarked against a traditional fixed-time traffic light cycle set at 60 s with a fixed green time of 20 s, a commonly used baseline in prior studies such as [13,16]. Table 11 summarizes the results of the analytical evaluation for the three considered density scenarios.

Under low demand (e.g., six vehicles), this is more than sufficient to clear the queue each cycle, producing a stable operation with an average uniform delay of 13.33 s per vehicle. However, at medium- and high-demand levels (e.g., 18 and 28 vehicles), the fixed-time system becomes overloaded since the service capacity of 15.38 (veh/min) is less than the arrival rates of 21.54 and 29.23 veh/min for the medium and high densities, respectively. In these settings, lines accumulate from cycle to cycle, and the average vehicle delay rapidly surpasses the red phase duration (more than 40 s for medium density, well over 60 s for high density), resulting in substantial congestion and possibly spillback onto upstream intersections. In addition, the throughput cannot exceed the capacity of 15.38 (veh/min) despite the increase in vehicle densities.

In the adaptive traffic light scenario, the effective green light time is calculated in real time using Equation (1) based on the number of detected vehicles. The green time is then adjusted by the traffic controller to consider the minimum/maximum operational bounds, simulation-based calibration, and cross-street fairness. For the low-density case, the green phase remains at 20 s to avoid overserving the approach, producing results identical to the fixed-time case (average delay of 13.33 s per vehicle). For the medium-density case, the adaptive algorithm increases the green phase to 28 s, resulting in a capacity of 21.54 vehicles per cycle, which exceeds demand and restores stable operation. The corresponding delay is reduced to 8.53 s per vehicle (78.7% reduction), and throughput increases by approximately 17.0% compared to the fixed-time baseline (15.38 vs. 18). For the high-density case, the green phase is extended to 38 s, near the 40 s cap set by the control algorithm. This yields a capacity of 29.23 vehicles per cycle, which is sufficient to serve the 28-vehicle demand without residual queues. The average delay is reduced to just 4.03 s per vehicle (93.3% reduction), and throughput increases by approximately 82.1% compared to the fixed-time baseline (15.38 vs. 28).

Overall, the results show that the proposed adaptive system eliminates oversaturation and significantly improves throughput in medium and high traffic volumes while maintaining performance under low demand. These results indicate that Passable improves traffic flow efficiency. On the safety aspect, the system’s ability to send immediate alerts about incidents contributes to reducing the risk of secondary accidents. This claim is supported by survey responses showing that 90% of drivers prefer to reroute if informed of an incident ahead, which aligns with findings in [33,34], highlighting the benefits of early warnings.

## 9. Discussion

This study contributes to intelligent transportation research by providing a realistic system that integrates incident detection, response, and alert communication to enhance traffic efficiency and road safety. The proposed system, Passable, is an intelligent traffic light system that combines deep learning-based incident detection, dynamic signal control, and wireless driver alerting. The technology demonstrated potential capabilities in addressing two interconnected urban traffic challenges—congestion and accidents—through immediate reaction and communication. Passable can detect accidents and congestion using trained YOLOv8 models and alter green light durations based on vehicle density. The results of YOLOv8 are consistent with the vision-based approaches in prior works utilizing earlier versions of YOLO, such as YOLOv5 [30,32]. Additionally, Passable can alert drivers to detected incidents, enabling them to take proper actions and reduce the impact and potential danger of these incidents.

Unlike most previous studies, which individually tackle accident and congestion tasks and ignore the fact of their interrelations [33,34], Passable adopted a comprehensive system approach that both supports detection tasks and implements proper reaction and communication strategies, reducing the risk of additional accidents and allowing better traffic flow. The adopted strategy is aligned with the recent efforts proposed by [35] but advances them further by integrating wireless driver alerts at the prototype level.

This strategy is vital in congested metropolitan areas, where accidents can immediately exacerbate traffic problems. Furthermore, the proposed prototype utilized a representative wireless communication technology, specifically Wi-Fi via ESP32 microcontrollers, which demonstrates the effectiveness of alerting nearby vehicles. This feature mitigates the impact of detected incidents and reduces the likelihood of potential secondary accidents, a frequently overlooked aspect in most existing intelligent traffic light systems.

The stakeholder survey and usability results confirmed that the system conformed to user expectations. Over 80% of participants reported a desire to use vehicle-based alert systems, and 90% favored rerouting if told of an event. Traffic officers emphasized the importance of automated solutions to support manual monitoring methods, which are now heavily reliant on patrols and CCTV networks.

While this work does not introduce a novel deep learning algorithm or optimization model, its contribution lies in the system-level design and operational integration of different technologies. Passable demonstrates how deep learning, IoT, wireless communication, and cloud platforms can be practically integrated in a deployable prototype to address multiple urban traffic challenges simultaneously—an aspect often missing in theoretical models or simulation-only studies.

Despite the demonstrated effectiveness of the proposed integrated traffic light system, there are some limitations. One of these limitations is related to the Passable hardware prototype that utilizes Wi-Fi, which is effective for demonstration but may not accurately reflect the robustness of more scalable communication technologies, such as DSRC, 5G, or LoRaWAN [16].

Another limitation is that vision-based detection can be affected by variations in light, weather conditions, and camera angles, thus reducing the detection accuracy. These limitations were noted in previous studies [21,23]. In addition, because our YOLOv8 models were trained on 720p CCTV footage, the model performance may suffer in scenarios with poor weather (e.g., rain, fog), low illumination (e.g., nightfall), or partial occlusions (e.g., huge cars obscuring the view). These characteristics, together with diminished visibility and contrast, can impair detection confidence and accuracy. To address this issue, pre-processing techniques, such as denoising and training with data that include adverse conditions and targeted augmentations, such as brightness variation and partial blurring, could be used to obtain more stable performance under adverse weather conditions and improve generalization. In locations with problematic conditions, the use of sensor fusion techniques that combine vision with complementary sensors (e.g., mmWave radar or LiDAR) could be considered to offer depth and motion cues that are less impacted by visual noise, thereby enhancing accuracy and minimizing misses in complicated urban situations.

Consistent with prior work [45,46,47,48], this study conducts a preliminary, proof-of-concept analytical evaluation of only the signal-control component using a fixed-time traffic light as a baseline. Fixed-time control was chosen as the baseline since it is widely used around the world, particularly in developing areas and smaller cities, due to its lower installation and maintenance costs compared to fully adaptive and actuated signal control systems [49]. In the future, an end-to-end performance evaluation of all Passable system components will be conducted. This evaluation will assess the impact of congestion or incident detection accuracy, as well as driver alerts, rather than focusing only on the signal timing component. In addition, the evaluation will compare the proposed adaptive signal control to other actuated and adaptive signal control strategies, providing a more rigorous benchmark than a fixed-time baseline.

While the current prototype was tested at a single intersection, the architecture was designed with modularity in mind. Each intersection operates as an independent edge node, equipped with its own camera, AI module, and communication unit. To scale the proposed system to multiple intersections, these nodes can be connected to a central management platform via a cloud or fog layer, enabling coordination, data aggregation, and inter-node communication. A hierarchical architecture could be used where each intersection has an edge node that runs CCTV-based detectors and a local green light timing algorithm, while fog or cloud nodes coordinate neighboring junctions (e.g., green-wave preemption for incidents). In addition, the dashboard’s city-level views make it an ideal supervisory layer for monitoring and policy modifications by decision-makers. Moreover, as the system is deployed in real-world scenarios, it will transition from using Wi-Fi to DSRC/5G/LoraWAN for increased resilience. Moreover, the AI models used can be enhanced by federated learning across junctions without sharing raw footage, thereby increasing efficiency and security. The alert protocol can be expanded by assigning unique identities to each intersection and sending location-specific messages to neighboring vehicles. This ensures that alerts are context-aware, avoiding overlap or confusion. Furthermore, advanced broadcast protocols employing event IDs and hop limits, as well as message scheduling and queuing controls, might be used to manage simultaneous alert messages across several junctions.

Integrating aerial monitoring based on UAVs and VLC-assisted V2X communication, as demonstrated in the work performed in [36], represents a promising enhancement to the Passable system. While our current implementation focuses on ground-based cameras and Wi-Fi communication, future deployments could leverage UAVs to provide overhead visibility of wider road segments and improve incident detection in occluded or congested areas. In addition, VLC can offer a low-latency, interference-resistant communication channel for short-range alerts using vehicles and traffic signals. However, VLC requires a clear line of sight, which may restrict its immediate implementation viability in mixed-traffic scenarios. Therefore, VLC could be used as a supplementary technology rather than a replacement for other communication technologies such as DSCRC, 5G, and LaRaWAN.

Furthermore, future work could focus on the specific issues presented by congested urban intersections, where channel congestion, interference, and “broadcast storm” phenomena can drastically limit system performance. To overcome these issues, edge-side message aggregation and de-duplication algorithms, as well as severity- and proximity-aware scheduling, could be utilized to guarantee that urgent and near-field hazards are prioritized in crowded situations. To further reduce redundant messages, several techniques could be implemented, including unique event IDs, spatial grouping for co-located alerts, adaptive rate limitation with exponential backoff, and TTL-based broadcast control. Additionally, intelligent vehicle-assisted relaying protocols could be used to increase coverage in urban canyons. Moreover, we could transition our prototype to V2X communication protocols such as DSRC, C-V2X, or 5G, which provide superior congestion management and quality-of-service assurances in high-density scenarios.

## 10. Conclusions

This paper presented Passable, a prototype for an intelligent traffic light system that utilized peer-to-peer communication between traffic lights and vehicles using ESP32. Although the developed prototype is a proof of concept, it offers strong insights about the applicability of the proposed intelligent traffic light system. To the best of our knowledge, Passable is the first traffic light control system that integrates accident detection, congestion detection, driver alerts, and centralized monitoring. The implemented prototype uses an ESP32 microcontroller with integrated Wi-Fi capabilities. Wi-Fi is used for peer-to-peer communication between the traffic light and vehicles, as well as between vehicles, enabling the dissemination of alert messages. The accident and congestion detection models utilize the YOLOv8 deep learning algorithm. The average incident detection accuracy of both models is 96%, effectively reducing traffic light waiting times, managing intersections, and sending alerts to vehicles using wireless technology. The prototype demonstrates the feasibility and effectiveness of using the IoT and computer vision in designing adaptive traffic light control systems. The design of such systems can contribute to improved traffic flow, enhanced safety, and reduced fuel consumption and carbon emissions by minimizing vehicle idling times. While our work currently focuses on a single intersection, it can be extended to include two or more neighboring intersections, which involves coordinating their cycles. This will improve the efficiency of the intelligent traffic light system, resulting in a smoother traffic flow throughout a larger area. The system’s capabilities can be further extended to detect emergency vehicles, such as ambulances. By prioritizing their passage, this technology can play a crucial role in saving lives and improving response times. The developed hardware prototype and analytical evaluation of Passable demonstrate improvements in reducing delays and increasing traffic throughput. However, they cannot fully capture the variability of real-world conditions. Thus, field validation is essential to assess robustness under diverse environments and scalability across multiple intersections. In addition, optimizing detection models and utilizing advanced communication technologies and protocols, such as DSRC, 5G, or LoRaWAN, could be part of our future work to test our prototype in real-world settings.

## Figures and Tables

**Table 1:** A comparative summary of adaptive traffic control systems.

Reference	Year	Accident	Congestion	Driver Alert	Prototype Type	AI Method	Communication	IoT Devices
[8]	2007	No	Yes	No	Simulation	No	Car-to-car (VANET), Controller-to-controller	Vehicles w/wireless devices, controller node
[9]	2010	No	Yes	No	Simulation	Genetic algorithm	No	No
[10]	2013	No	Yes	No	Hardware	Rule-based logic	GSM, GPRS	RFID controllers and tags
[11]	2018	No	Yes	No	Hardware	Image processing	Cloud (ThingSpeak)	Raspberry Pi, ultrasonic sensors, camera
[12]	2020	No	Yes	No	Hardware	No	Wireless	Arduino, IR, and magnetometer sensors, camera
[13]	2021	No	Yes	No	Real intersection	YOLOv4, XGBoost	No	No
[14]	2022	No	Yes	No	Simulation	Multi-agent system	RFID or DSDR	Infrared sensors, traffic lights w/DSDR/RFID
[15]	2023	No	Yes	No	Simulation	Multi-agent reinforcement learning	No	Camera
[35]	2023	Yes	Yes	Yes	Simulation	DBSCAN	Wireless	IR and smoke sensors, microphone, HC-12
[16]	2024	No	Yes	No	Hardware	Optimized Tiny YOLOv3	IEEE 802.15.4 DSME, LoRaWAN	Raspberry Pi, camera, IEEE 802.15.4 and LoRa transceivers
[17]	2024	No	Yes	No	None	YOLOv8	No	No
[36]	2024	No	Yes	No	Simulation	YOLOv4	VLC	UAV
Passable	2025	Yes	Yes	No	Hardware	YOLOv8	IEEE 802.11 (Wi-Fi)	Arduino, camera, ESP3 microcontroller

**Table 2:** Participants’ demographic information.

Questions	Answers	No. Responses	Percentages
1. What is your age group?	Between 18 and 24 years	325	73.6%
	Between 25 and 45 years	76	17.2%
	Above 45 years	41	9.2%
2. What region in Saudi Arabia do you live in?	Mecca region	380	85.9%
	Riyadh region	26	5.9%
	Other Saudi Arabia regions	36	8.2%
3. What is your occupation?	Student	298	67.4%
	Employed	71	16.1%
	Unemployed	73	16.5%
4. How frequently do you drive your car?	I don’t drive	236	53.4%
	About 0–2 times a week	43	9.7%
	About 3–5 times a week	47	10.6%
	More than 5 times a week	116	26.3%

**Table 3:** Participants’ responses to survey questions.

Questions	Answers	No. Responses	Percentages
1. Do you suffer from traffic congestion at traffic lights?	Yes	402	90.9%
	No	40	9.1%
2. Have you ever waited for a red light while the other traffic lights at the intersection had no cars waiting?	Yes	382	86.5%
	No	60	13.5%
3. What is the maximum number of times a traffic light opened and closed before you were able to pass it?	1 time or less	60	13.5%
	2 times	184	41.6%
	3 times	137	31.1%
	4 times	34	7.6%
	5 times or more	27	6.2%
4. Have you ever been involved in a secondary accident?	Yes	48	10.9%
	No	246	55.7%
	No, but I know someone who has	148	33.4%
5. Have you ever experienced an accident caused by sudden acceleration or a sudden stop when a traffic light changed its color?	Yes	89	20.2%
	No	235	53.1%
	No, but I know someone who has	118	26.7%
6. Can you estimate the average time you require to pass a traffic light?	Less than 5 min	218	49.3%
	Less than 10 min	148	33.4%
	Less than 15 min	50	11.4%
	16 min or more	26	5.9%
7. If you were aware of an accident or severe congestion in the direction you were heading, which would you prefer?	Change route	398	90%
	Continue on the same route	42	10%
8. Are you willing to use a vehicle equipped with communication technology to receive notifications about congestion or accidents?	Yes	363	82.1%
	No	9	2.1%
	Maybe	70	15.8%
9. Which of the following types of alerts do you prefer in your vehicle (select all that apply)?	Voice alert	286	64.8%
	Text message	154	34.9%
	Light alert	279	63%
10. Would an AI-powered system help save time, prevent accidents, reduce traffic congestion, and make better use of resources?	Yes	354	80.1%
	No	6	1.5%
	Maybe	82	18.5%

**Table 4:** Summary of training experiments for congestion detection.

Experiment	Epochs	Batch Size	Accuracy	mAP50	Recall	Precision
1	150	16	95%	97.3%	93.7%	93.4%
2	150	32	96%	97.5%	94.3%	91.8%
3	200	16	94%	97.4%	93.5%	94.2%
4	200	32	94%	97.3%	93.4%	93.7%
5	250	16	95%	97.2%	93.8%	93.3%
6	250	32	94%	97%	94.4%	91.5%

**Table 5:** Summary of training experiments for accident detection.

Experiment	Epochs	Batch Size	Accuracy	mAP50	Recall	Precision
1	150	16	91%	95.3%	93.1%	91.7%
2	150	32	97%	97.6%	91.7%	97.6%
3	200	16	93%	96.6%	90%	97%
4	200	32	92%	96.6%	97.1%	91.3%
5	250	16	96%	98.2%	96.4%	95%
6	250	32	95%	96.6%	93.9%	95%

**Table 6:** Model testing results.

Testing Dataset	Accuracy	mAP50	Precision	Recall	F1-Score
Congestion dataset	95%	96.5%	93.5%	91.8%	93%
Accident dataset	97%	91%	88%	96.6%	92%

**Table 7:** Traffic density classification.

Classifier	Meaning	Definition
Low	Only a few cars	0–8 cars
Medium	Slightly filled street	9–20 cars
High	Filled street	>20 cars

**Table 8:** Task success measurement criteria.

Task	Task Description	Completion Time (s)	Clicks
1	Sign up to the dashboard	25	6
2	Log in to the dashboard	5	3
3	Retrieve total number of traffic accidents	4	0
4	Retrieve total number of traffic accidents in the western area	5	1
5	Retrieve total number of traffic congestion incidents	3	0
6	Retrieve congestion incidents on King Abdulaziz Road	7	2
7	Identify the date with the highest number of incidents this year	4	1
8	List the top three most congested intersections in the city	4	0

**Table 9:** Average time (s) taken for each task.

Task	User 1	User 2	User 3	User 4	User 5	Average Time
1	27	35	31	37	36	33.2
2	6	8	10	9	8	8.2
3	4	5	7	7	6	5.8
4	13	20	17	21	15	17.2
5	3	4	5	3	5	4
6	7	9	8	7	8	7.8
7	5	7	7	6	6	6.2
8	6	7	5	6	5	5.8

**Table 10:** Average number of clicks for each task.

Task	User 1	User 2	User 3	User 4	User 5	Average Number of Clicks
1	6	6	6	6	6	6
2	3	3	3	3	3	3
3	0	0	0	0	0	0
4	2	5	3	4	3	3.4
5	1	4	4	3	2	2.8
6	1	4	5	7	2	3.8
7	2	3	2	1	1	1.8
8	0	1	3	2	0	1.2

**Table 11:** Analytical performance comparison between fixed-time and adaptive control, showing percentage improvements in delay and throughput. Capacity is computed using Equation (4), delay is calculated using Equation (5), traffic throughput is calculated using Equation (6), and improvements are calculated from Equations (7) and (8).

Traffic Density	Demand (veh/min)	Green Time (s) Fixed/Adaptive	Capacity (veh/Cycle) Fixed/Adaptive	Avg Delay Time (s/veh) Fixed/Adaptive	Throughput (veh/min) Fixed/Adaptive	Delay Reduction (%)	Throughput Increase (%)
Low	6	20/20	15.38/15.38	13.33/13.33	6.00/6.00	0.0	0.0
Medium	18	20/28	15.38/21.54	>40.0/8.53	15.38/18.00	78.7	17.0
High	28	20/38	15.38/29.23	≫60.0/4.03	15.38/28.00	93.3	82.1

## Data Availability

The original data presented in the study are openly available in the TrafficObjectDetectionDataset5 at [https://universe.roboflow.com/inlights-s5jdz/traffic-sfpps/dataset/5, accessed on 20 May 2025], TrafficObjectDetectionDataset1 at [https://universe.roboflow.com/inlights-s5jdz/traffic-sfpps/dataset/1, accessed on 20 May 2025], and CarCrashDetection at [https://universe.roboflow.com/mada-study/car-crash-detection-c9xlj/dataset/1, accessed on 20 May 2025].
